# A Review on Thermophotovoltaic Cell and Its Applications in Energy Conversion: Issues and Recommendations

**DOI:** 10.3390/ma14174944

**Published:** 2021-08-30

**Authors:** Mansur Mohammed Ali Gamel, Hui Jing Lee, Wan Emilin Suliza Wan Abdul Rashid, Pin Jern Ker, Lau Kuen Yau, Mahammad A. Hannan, Md. Zaini Jamaludin

**Affiliations:** 1 Institute of Sustainable Energy, College of Engineering, Universiti Tenaga Nasional, Kajang 43000, Malaysia; mansur.gamel@uniten.edu.my (M.M.A.G.); Hannan@uniten.edu.my (M.A.H.); 2 Department of Electrical and Electronics Engineering, College of Engineering, Universiti Tenaga Nasional, Kajang 43000, Malaysia; LHJing@uniten.edu.my; 3 Institute of Power Engineering, College of Engineering, Universiti Tenaga Nasional, Kajang 43000, Malaysia; Emilin@uniten.edu.my (W.E.S.W.A.R.); Mdzaini@uniten.edu.my (M.Z.J.); 4 State Key Laboratory of Modern Optical Instrumentation, College of Optical Science and Engineering, Zhejiang University, Hangzhou 310027, China; 0621072@zju.edu.cn

**Keywords:** thermophotovoltaic, InGaAs, GaSb, narrow bandgap, performance

## Abstract

Generally, waste heat is redundantly released into the surrounding by anthropogenic activities without strategized planning. Consequently, urban heat islands and global warming chronically increases over time. Thermophotovoltaic (TPV) systems can be potentially deployed to harvest waste heat and recuperate energy to tackle this global issue with supplementary generation of electrical energy. This paper presents a critical review on two dominant types of semiconductor materials, namely gallium antimonide (GaSb) and indium gallium arsenide (InGaAs), as the potential candidates for TPV cells. The advantages and drawbacks of non-epitaxy and epitaxy growth methods are well-discussed based on different semiconductor materials. In addition, this paper critically examines and summarizes the electrical cell performance of TPV cells made of GaSb, InGaAs and other narrow bandgap semiconductor materials. The cell conversion efficiency improvement in terms of structural design and architectural optimization are also comprehensively analyzed and discussed. Lastly, the practical applications, current issues and challenges of TPV cells are critically reviewed and concluded with recommendations for future research. The highlighted insights of this review will contribute to the increase in effort towards development of future TPV systems with improved cell conversion efficiency.

## 1. Introduction

A TPV system converts thermal radiations from various heat sources such as the combustion of fuels, industrial waste heat, concentrated solar or nuclear energy into electricity. For example, fossil fuels are the main energy source for world-wide energy consumption. However, they are non-renewable resources that will deplete over time due to impulsive mining. Panayiotou et al. [1] has estimated that 370.41 TWh/yr of waste heat is generated from European industries in 2017. This massive amount of waste heat generation has led to a worldwide concern on the global environmental impact and a quest for efficient use of waste heat in the industries. Therefore, there is an urgent need to explore alternatives to improve waste heat recycling and energy conversion efficiency to minimize the reliance on fossil fuels. In this regard, a thermophotovoltaic (TPV) system appears to be a potential candidate to meet these requirements (The meaning of all short names are available in Appendix A). Moreover, the flexibility of converting various heat energy sources such as solar, nuclear, chemical combustion, and waste heat into high electrical power density broadens the TPV application ranging from micro-scale to large-scale TPV generators [2]. For instance, a worldwide potential of 3.1 GW electricity generation using TPV system in steel industry (>1373 K) was estimated by Fraas et al. [3].

In comparison to a solar photovoltaic system, a TPV system works for a longer operation time at a lower radiator heating temperature [4]. A TPV system consists of four main devices: a generator to provide heat energy from the fuel combustion process, a radiator to translate the heat energy into an emission spectrum, a filter to coordinate the emission spectrum to a TPV cell, and lastly a TPV cell to convert the photon radiation into electrical energy [5]. A comprehensive analysis has been conducted in each component of the TPV system to enhance the overall performance. Particularly, the TPV cell, which converts the photon radiation directly into electricity is the core component that contributes to the overall TPV system performance [6]. Therefore, this review comprehensively studied narrow bandgap TPV cells namely the gallium antimonide (GaSb), indium gallium arsenide (InGaAs) and a few other potential narrow bandgap materials such as germanium (Ge), indium arsenide (InAs), indium gallium arsenide antimonide (InGaAsSb), indium arsenide antimonide phosphide (InAsSbP), and indium gallium arsenide antimonide phosphide (InGaAsSbP) TPV cells. Their respective cell performances, improvements and challenges will be highlighted.

Over the last three decades, research on various parts of the TPV system has received tremendous attention. The advantages of noiselessness, high reliability, mechanical stability without moving parts, and a large power density, make TPV suitable for a vast range of terrestrial and space applications. Recently, numerous review papers have been published. In 2014, Ferraria et al. [7] presented and discussed a critical review of the TPV prototypes. In the next year, Daneshvar et al. [8] reviewed the development of all main components, discussed the fundamental and technical challenges facing commercial adoption of TPV and prospects of TPV. Mustafa et al. [9] summarized the progress of combustion-driven thermoelectric (TE) and TPV power generation systems for the years 2000–2016. Datas and Martí [10] reviewed the state of the art and historical development of TPV for space application along with the main competing technologies. Tain et al. [11] reported the recent progress of near-field and far-field radiative heat transfer, various design structures of metamaterials and their properties, and focused on the exploration of tunable radiative wavelength selectivity of nano-metamaterials. More recently, in 2019, Sakakibara et al. [12] reviewed the state of the art of radiator and presented a systematic approach for assessing radiators. A recent paper from Rashid et al. [13] has highlighted the recent development of TPV for waste heat harvesting application and investigated the potential implementation in coal-fired thermal power plant. Furthermore, Burger et al. [14] studied numerous decades of experimental TPV works and compared the energy-conversion of different systems with respect to experiment-specific thermodynamic limit. Based on the research gap, a review on the comparison of performance parameters of different TPV cell materials and their respective improvement and potential are yet to be conducted. Therefore, this paper focuses on the TPV cell, which is the main component in the TPV system. Furthermore, the comprehensive review on various TPV cells contributes to the understanding of the decades of advancement, future prospects, and applications of TPV cells.

The structure of the paper is as follows. Section 2 presents the overall TPV system and the analytical aspects involving the TPV cell conversion. Section 3 summarizes the methods to fabricate TPV cells. Section 4, Section 5 and Section 6 review GaSb, InGaAs, and other narrow bandgap TPV cells, respectively. The engineering applications are discussed and presented in Section 7. In Section 8, we provide some current challenges and recommendations for future research prospects in this field. Even though not all the issues and challenges in TPV systems can be immediately addressed, recommendations are provided on the current issues which can be potentially resolved. This review will provide a clear and accessible guidance on a complete TPV system and a thorough literature study of TPV cells together with the development, performance, improvement, and the current issues with concrete recommendations.

## 2. TPV System Overview

**Figure 1:**
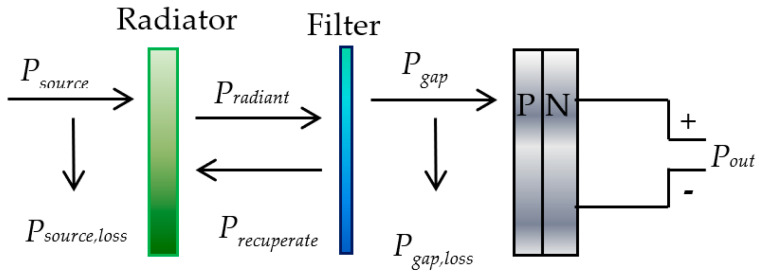
Schematic diagram of an overall TPV system.

A TPV system converts radiant energy from a generator into electrical energy using TPV cells. Figure 1 shows the TPV system which includes a generator, a radiator, a filter and an array of TPV cells [7]. The generator produces power from various heating sources (*P_source_*) to the radiator with certain heat loss (*P_source,loss_*). Next, the radiator generates the radiant power (*P_radiant_*) to the PV cells via the filter. The filter then narrows the emission band from the radiator. The filtered radiated energy from the radiator should exceed the bandgap of PV cells with the bandgap power (*P_gap_*), whereas losses (*P_gap,loss_*) are induced by the photons with lower energy than the bandgap of PV cells. These photons are recuperated to the radiator (*P_recuperate_*) to conserve heat and reduce *P_source_* at the required radiator temperature. The output power (*P_out_*) at PV cells is measured through the optical-to-electrical signal conversion process [7].

A generator is a heat-driven source for TPV system with a typical working temperature range from 1000 to 2000 K [9,15]. This generator can be concentrated solar radiation, radioisotope thermal generator, combustion of hydrocarbon fuels or industrial waste heat [16,17]. Solar radiation produces the highest temperature among the generators. Nevertheless, this high temperature is attained only at the AM 0 condition. Subsequently, the installation angle of TPV cell to harvest solar radiation at AM 1.5 condition should be optimized to achieve higher radiation energy efficiency. Apart from solar radiation and radioisotope, liquid and gas fuels such as oil, butane, propane, methane, and hydrogen are employed to drive a generator [18,19,20,21,22,23,24,25]. Various TPV combustor–regenerator systems for electric vehicles have been studied both theoretically and experimentally [26]. The combustion system was optimized via studying the thermo-fluid dynamic model. Chamber geometry, fuel injection, and mass flow rate were found to be dominant parameters. Furthermore, the heat exchanger optimization was obtained by substituting the matrix material with a ceramic material that is lighter than metal and with a greater heat capacity to store a high amount of energy. Additionally, ceramic matrix was able to obtain a greater porosity and thus a greater surface for the heat exchange with a reduction of volume and weight. Colangelo et al. [27] designed and tested various TPV combustors and heat recovery systems for different testing conditions. It was found that a rotary heat exchanger is an optimal design since it is very compact and has higher effectiveness in comparison with other types of regenerators with the same number of transfer units. Furthermore, the study developed a model which accurately predicts the performance of the heat exchanger, taking into account two different values for the physical properties (such as thermal conductivity, heat capacity) for the hot and cold sides of the regenerator.

A radiator emits electromagnetic energy by translating heat from generators into an emission spectrum to provide appropriate receiver cell sensitivity [22]. Selective radiators such as silicon carbide (SiC), tungsten (W), W-SiO_2_ rare-earth oxide, and photonics crystal (PhC) provide narrow spectral range emission by enhancing in-band radiation and suppressing out-of-band radiation [28,29,30,31,32,33,34]. There are two significant types of selective radiators, namely the rare-earth oxide radiator and the broadband radiator [35]. Bitnar et al. [20] reported that the maximum emissivity of ytterbia and erbia radiators is 0.85 at a photon energy of 1.27 eV and 0.82 at a photon energy of 0.80 eV, for temperatures 1735 K and 1680 K, respectively. The bandgap of the selective radiator must be higher than the bandgap of TPV cell to minimize the build-up of recombination for higher electrical energy conversion efficiency purpose. Therefore, Si (1.1 eV) cell is suitable for the ytterbia radiator while GaSb (0.7 eV) TPV cell is suitable for the erbia radiator. A promising radiator has been achieved by vacuum plasma spray coating of rare earth oxides on intermetallic alloy MoSi_2_. The radiator can operate in an oxygen-containing atmosphere at a temperature of 1873 K, which is highly thermal-shock stable and shows good selective-emitting properties [36].

Broadband radiator establishes the emission across a wide range of wavelength for temperature range between 1000 to 2000 K [32,37]. Examples of broadband radiators are alumina, zirconia, magnesia, silica, yttria, and more, which possess a major challenge in low thermal shock resistance and low emissivity [38]. SiC with a minimum bandgap of 2 eV [39] has proven to be a suitable TPV radiator which can endure high melting point and high emissivity close to a 0.9 µm wavelength at an operating temperature up to 1923 K [38,40]. A SiC porous superadiabatic radiant burner (radiator) was experimentally proposed for TPV system, achieving radiator efficiency up to 32% and a system output power of 5–10 W [41]. A broadband radiator shows advantages over a selective radiator due to the simplicity in fabrication, higher durability and less labor-intensive [35]. Nevertheless, these advantages are attained at the cost of lower TPV system efficiency and power density as compared to selective radiator [23]. Gentillon et al. [42,43] experimentally characterized and analyzed a design of a porous media combustion-based thermophotovoltaic reactor with controlled radiant emission using yttria-stabilized zirconia/alumina composite (YZA). It was found that the erbia coating on YZA foam increases the emissivity by ~10%.

A filter is located in between the radiator and the TPV cell to spectrally filter the emission from the radiator, it is matched to the bandgap of TPV cell to block the energy of photons that is lower than the energy bandgap of a TPV cell [7]. The TPV cell performance is optimized by selectively filtering the thermal radiation depending on the radiator temperature and bandgap of TPV cell. This is to promote photon recycling and to improve system conversion efficiency [20,44]. Catchpole et al. [45] demonstrated that 99% of photon energy sit above the TPV bandgap using a highly idealized filter with 0.7 V applied voltage. Tong et al. [46] proposed on the utilization of intermediate frequency filter and photon recycling back to the radiator. Interference filter or dielectric filter is realized as a multilayer stack which can be deposited over the cell or to be placed between the radiator and the cell. The interface creates a low pass filter that cuts at a specific wavelength [8,47].

**Figure 2:**
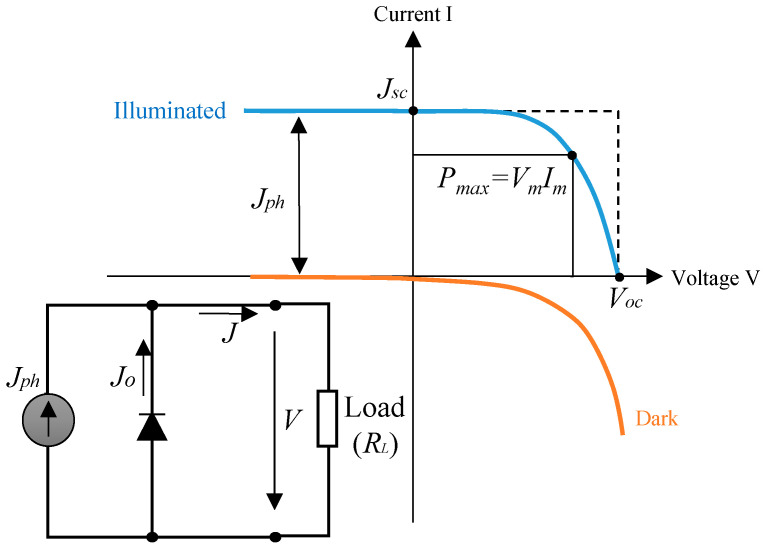
The equivalent circuit and IV characteristics of dark cell when *J_ph_* = 0.

TPV cell converts the photon radiation into electricity and share similar principal as PV cell. The total current density flow through the load, *J* in Figure 2 is expressed as [4]:

(1) $$J=J_{o}\;\left(e^{\frac{e_{o}V}{kT_{cell}}}-1\right)-J_{ph}$$

In the dark condition, there will be no current generated by per unit area, thus the *J_ph_* is equal to zero. The relationship between the current and voltage is expressed using Shockley diode equation, as shown in Equation (1). Figure 2 shows the current-voltage (*IV*) characteristics of the dark cell when the *J_ph_* is zero. When the cell is illuminated, each photon above the bandgap (*hV_g_*) will contribute to one elementary charge (*e_o_*), generating the *J_ph_*. The *IV* curve will therefore be shifted to quadrant IV where information about maximum current density *(J_m_), V_m_* and maximum power can be extracted. The maximum power output is utilized by the external load, *R_L_*.

All radiated photons must be fully absorbed and converted into photocurrent to achieve a maximum *J_ph_*. In this context, the external quantum efficiency (*EQE*) defines the photon-to-photocurrent conversion efficiency of a TPV cell. *EQE(λ)* as a function of *λ* describes the probability of a photon with wavelength *λ* absorbed by the cell which generates electron-hole pair that will be collected at the terminal. *EQE(λ)* represents the functionality of p-n junction in detail as it considers both the reflection and absorption coefficient of the incident photon as well as the collection of the minority carriers. *EQE* can be solved from *J_sc_*, which is expressed as a function of incident photon flux *Φ*(λ) given by:

(2) $$J_{sc}=e\;\int_{0}^{\lambda_{GAP}}\Phi \left(\lambda \right)\cdot EQE\;\left(\lambda \right)\cdot d\left(\lambda \right)\;$$

where *d*(λ) is the penetration distance [7]. TPV radiator with temperatures range from 1473 to 2073 K has a low photon emission at 1000 nm wavelength. For emitted photons with energy lower than the bandgap where absorption is not optimum, a selective filter can be used to redirect source and hence improve the efficiency.

The efficiency of TPV system, $\eta_{TPV}$ to convert radiated heat to electrical power is expressed as follow [48]:

(3) $$\eta_{TPV}=\eta_{sp}\eta =\frac{P_{out}}{P_{radiant}}$$

where $\eta_{sp}$ is the spectral efficiency of the radiator which is presented as:

(4) $$\eta_{sp}=\frac{\int_{0}^{\lambda_{c}}E\left(\lambda \right)\cdot \varepsilon \;\left(\lambda \right)\cdot d\lambda \;}{\int_{0}^{}E\left(\lambda \right)\cdot \varepsilon \;\left(\lambda \right)\cdot d\lambda \;}$$

where *E*(λ) is the blackbody spectrum and *ε*(λ) is the spectral emissivity of the proposed selective radiator metamaterial.

In this regard, it is observed that there are two main scopes which require further study and investigation. Firstly, it is crucial to improve the heat transfer between the heat source and the TPV cell. This can be done by increasing the emissivity and the use of suitable type of selective radiator. The second approach is to improve the *EQE* of the cell, which leads to higher cell efficiency.

## 3. TPV Cell Fabrication

There are two methods of TPV cell fabrication, namely non-epitaxial and epitaxial methods. Non-epitaxial growth can be sub-categorized into two: diffusion method and ion implantation method. Diffusion method is commonly used to fabricate GaSb TPV cell [49,50,51] and InGaAs [52]. The conventional diffused GaSb-based TPV cell is manufactured in a pseudo-closed box (PCB) with the diffusion of Zn particle into Tellurium-doped single-crystal GaSb substrate [53]. Parameters studied on the diffusion profiles are temperature, diffusion time and precision of control for the depth of p-n junction. Tang et al. [49,50] presented a closed-quartz-tube for the diffusion process where Zn-Ga alloy is proven to be a suitable source that can suppress the formation of high concentration surface region in Zn profile with a lower fabrication cost.

Ion implantation method is the most suitable method to perform selective doping, as the spatial distribution of dopant atoms can be more precisely defined [54]. However, the use of ion implantation introduces undesirable damage to the lattice crystal structure due to the high annealing temperature [54,55,56]. The formation of junctions appears to be more difficult than diffused junctions [57,58]. This causes a non-uniform p-n junction formation due to different thicknesses of the active region. Rahimi et al. [59] demonstrated that the Be-implanted GaSb exhibits similar performance to the MBE-grown GaSb TPV cell. To achieve this, the implanted dopants on the semiconductor substrate must undergo a rapid thermal annealing (RTA) process where the cell is exposed to a high temperature to remove the implant-induced damage and therefore achieving a higher shunt resistance [55,60]. It is highlighted that inadequate isolation is produced from the ion bombardment process due to small intrinsic resistivity of InGaAs material [61]. The main limitation of the non-epitaxial growth method is the high front and back surface recombination which reduce the photocurrent collection. Several studies proposed an advance growth method that combined epitaxial and diffusion method [62,63]. The main advantage of the combined growth technique is to create a device with low surface recombination and low defect density.

Epitaxy is a process of depositing crystalline on a substrate that acts as a seed crystal, which is favorable for achieving a better cell performance with the advantages of better purity control, thickness control and doping level control. The epitaxy can be categorized into three different mediums: liquid, solid and vapor. Liquid phase epitaxy (LPE) is the deposition of liquid phase single-crystalline either in the solution or melt form on a substrate crystal below the melting temperature of deposited materials [64]. Most TPV cell structures are initially fabricated using LPE method due to the simplicity of the process. TPV cells grown by LPE method suffer from very high lattice mismatch [65] and poor thickness control, which affect the cell efficiency. Epitaxial lateral overgrowth (ELOG) is introduced to solve the mismatching issue in heteroepitaxy [66,67]. It is worth highlighting that ELOG blocks mismatching threats from substrate [68]. Cheetham et al. [69] described a well-established low bandgap structure using LPE growth method with InAs_0.62_Sb_0.14_P_0.24_/Ga_0.03_In_0.97_As_0.83_Sb_0.14_P_0.03_ on InAs substrate. In 2015, Krier et al. [65] developed a InAs_0.61_Sb_0.31_P_0.26/_InAs p-n junction with 0.32 eV bandgap using the LPE method. Hence, LPE is a promising technique to produce a larger size single crystal with high-quality binary, ternary and quaternary TPV structures at relatively low growth temperature [70]. Despite the simplicity and low cost of LPE method, vapor phase epitaxy (VPE) is capable of producing cells with better crystal quality and higher performance.

VPE can be subcategorized into molecular beam epitaxy (MBE), metal-organic vapor phase epitaxy (MOVPE) and plasma-enhanced chemical vapor deposition (PECVD). MOVPE method was introduced for the growth of vapor phase III-V compound semiconductor materials, such as GaSb or InSb, on different types of substrate surfaces [71]. MOVPE is suitable for numerous commercialized low bandgap devices, with the advantages of a low reactor downtime, ease of maintenance, easy scalability for multi-wafer deposition, as well as more stable and controllable growth rates. In addition, MOVPE is more suitable for the growth of high-quality InP buffer and cladding layers due to lower arsenic contamination [72]. MOVPE is often use in high-quality materials and more complex structures with higher interface quality [73]. Material quality can be significantly improved by all parameters which reduce atomic surface diffusivity, such as decreasing growth temperature, increasing growth rate, and substrate miscut angle [74]. TPV cell technology is approaching 30% cell efficiency at 300 K cell temperature due to the gradual improvement in the MOVPE manufacturing process [75]. The experimental data of a simple Zn-diffused GaSb structure as compared to the complex MOVPE structure has proven that MOVPE structure had better performance with a maximum *FF* of 75% as compared to 70% with Zn diffusion structure [76]. One of the essential advantages of MOVPE method is the wide selection range of substrate materials. Most work in the MOVPE-fabricated TPV cells was conducted to improve the density of mismatching using cheaper substrate materials. For an InGaAsSb TPV cell, GaAs substrate is a better option compared to GaSb as the cost is cheaper with a higher potential to be commercialized [77]. Despite high lattice dislocation (7%) between GaSb p-n junction and GaAs substrate, the structure was improved by shifting the active junction away from the substrate material using selective epitaxy technique to create a buffer layer. The output power of GaSb/GaAs cell is only 30% lower than homojunction GaSb cell, under the same illumination condition. In another study, InGaAsSb on GaAs exhibited similar dark current-voltage characteristic with that on GaSb substrate. Furthermore, the $J_{SC}$ and $V_{OC}$ of fabricated structure are comparable with GaSb-based structure under illumination from 1073 K silicon nitride radiator [78]. In another study, Lu et al. [79] reported the use of a novel metamorphic buffer layer to suppress the threading dislocations originating from the large lattice-mismatch of InGaAsSb on GaAs substrate, which included the interfacial misfit arrays at the GaSb/GaAs interface and strained InGaSb/GaSb multi-quantum wells acting as dislocation filtering layers.

MBE utilizes an ultra-high vacuum (UHV) with a low deposition pressure in the chamber (lower than 10 Torr). This technique provides a clean growth environment, higher purity, precise control of the beam fluxes and growth condition by changing the nature of the incoming beam. The MBE method has the advantage of generating complicated doping profiles due to the flexible control of the dopants. The MBE method provides promising fundamental device parameters such as low ideality factor (*n* = 1.0) and low dark current of 6 × 10^−5^ A. However, a GaSb structure grown over a large area is challenging due to the difficulty of finding an epi-ready substrate, non-uniform native oxide desorption, and shunt defect formation. A key advantage of using the MBE method to grow TPV cell is the generation of a higher $V_{oc}$ when compared to the MOVPE and LPE methods [59,80]. Table 1 provides a summary of characteristics, advantages and disadvantages for different types of TPV cell growth methods.

## 4. GaSb-Based TPV Cell

In this section, a comprehensive study on GaSb-based TPV cell in terms of their historical development, cell performance, performance improvements and challenges are thoroughly discussed.

### 4.1. Introduction

GaSb is a III-V binary semiconductor compound. It is considered as one of the ideal semiconductor materials for TPV applications with temperature source ranging from 1300 K to 1500 K. Due to its low bandgap energy ($E_{g}$), which spectrally matched with the medium blackbody temperature, it produces an excellent quantum efficiency of greater than 90%, especially at IR wavelength up to 1800 nm [117]. A few advantages of the GaSb semiconductor material compared to the other conventional materials such as Si, Ge are the direct bandgap properties with 0.72 eV energy at 300 K, and the cell performance is less affected at higher operating temperature [118]. Historically, in 1989, Dr. Lewis Fraas invented a GaSb photocell using Zn diffusion method as a booster cell, mechanically stacked under GaAs material for concentrator solar cell application [119], with a world-record energy conversion efficiency of 35% [53,120]. Later in 1995, there is a major breakthrough of an electric generator using GaSb TPV cells with a hydrocarbon burner patented by Fraas and associates [121]. In addition, JX Crystal Inc. commercialized the “Midnight Sun” TPV stove for residential heating application in 1995 [21]. Morrison and Seal successfully built a prototype of the world’s first TPV-powered automobile named “Viking 29” which utilizes 20 series connected GaSb cells with over 41 W power generation [122,123]. In the 2000s, Stollwerk et al. [124] conducted the first simulation on GaSb TPV cell using two-dimensional (2-D) numerical solar cell simulation program. The advancement in material widens the opportunity for the researchers to explore various TPV applications.

Nowadays, the GaSb TPV cells are technologically matured and commercially available. Research efforts towards maximizing the conversion efficiency increase tremendously in the recent years. In 2014, GaSb TPV cell is used to generate electricity from waste heat in the steel industry which generates 3.1 GW of power under 1673 K [3]. Recently, an evaluation study of GaSb TPV cell performance at low-, mid- and high-waste heat temperature was conducted by researchers in Turkey targeting the potential waste heat location in the Turkish Industrial Sector [125,126,127]. The GaSb-based TPV cell is currently an active research area and expected to be continually developed in the near future. Taking advantage of its infrared radiation sensitivity, the GaSb TPV cell has great potential for waste heat energy harvesting. Significantly, the research on GaSb for TPV applications is advancing rapidly since the industrial processing and activities have been constantly emitting a huge amount of potential waste heat to be recovered.

### 4.2. Performance of GaSb-Based TPV Cell

The early GaSb photovoltaic cell structure that is sensitive to the photons in the infrared region up to 1.8 µm was invented and patented in 1988 by McLeod et al. (see US 4,776,893 [128] patent) and Fraas et al. (see US 5096505 [129] and US 5091,018 [130] patent). The cell was intentionally designed to be used as an infrared booster cell stacked tandemly under GaAs solar cell for concentrated sunlight solar application. Since the early invention, the performance of a single GaSb cell under 100 suns concentrated light intensities was recorded with an FF of 71.3%, $V_{oc}$ of 0.48 V, and $J_{sc}$ of 2702 mA/cm^2^ [130]. Additionally, around a 7% conversion efficiency of GaSb solar cell under the full sunlight spectrum range was recorded through the fundamental characterization studies [53]. Therefore, GaSb cell provides a good electrical performance for infrared radiation capture.

As aforementioned, a major breakthrough in GaSb TPV cell development was made in 1995 by JX Crystal Inc (Fraas and associates). Fraas et al. [131] has developed a prototype of a combined home furnace-TPV generator and reported a much higher power output. Under a SiC blackbody radiator at 1523 K, over 1 W/cm^2^ of power output density per cell was demonstrated. Later on in 2014, Fraas et al. [3] projected a TPV planar module performance for steel industry application at 1400 K and 1500 K blackbody temperature with 20% efficiency and output power of 1.8 W/cm^2^. Subsequently, a cell efficiency of 29% with an electric power of 1.5 W/cm^2^ for a single GaSb cell measured under 1548 K radiator temperature was reported. Several studies benchmarked their GaSb TPV cells performance with a fabricated device by JX Crystals Inc. For example, Tang et al. [49] demonstrated a new Zn diffusion process for GaSb-based TPV cells. The cell demonstrated a lower performance as compared to JX Crystals Inc device due to the higher series resistance at both front and back electrodes. In contrast, Ni et al. [117] compared an epitaxially grown, thin-film GaSb TPV cell and reported higher theoretical *IQE* performance in comparison to the device fabricated by Fraas et al. [94]. It is highlighted that the epitaxial GaSb TPV can reach approximately 96% of *IQE* when optimizing the emitter and base layer thickness to 0.22 and 10 µm, respectively. In the same vein, Rashid et al. [5] investigated the effect of emitter layer region and compared the GaSb TPV cell performance with a Zn-diffused commercialized JX Crystals Inc device. Based on their simulation result, an efficiency increment from 4.70 to 7.88% was recorded with optimized emitter thickness and doping concentration under AM 1.5 illumination condition.

Several studies have made comparisons between epitaxially grown and non-epitaxially grown (ion-implantation and diffusion) GaSb TPV cells. For example, Martin et al. [132] and Rahimi et al. [59] compared the performance of epitaxial and non-epitaxial GaSb TPV cells. Rahimi emphasized that an annealing process removed implant-induced damage crystal on GaSb devices, resulting in comparable device quality with epitaxially grown GaSb TPV cell. The performance of both epitaxial and non-epitaxial GaSb TPV cells are comparable and suitable to be employed for TPV generators. This statement was supported by Schlegl et al. [76] who demonstrated a slightly better performance of the MOVPE-grown GaSb TPV cell when compared to the Zn-diffused cell. Nevertheless, the method for the epitaxially grown device is more sophisticated and expensive. To date, the GaSb cell fabricated by Zn diffusion method is favorable in various TPV applications due to the simplicity of the fabrication technique. The performance of various single GaSb cells fabricated by Zn diffusion method are summarized in Table 2. However, previous experiments involved various radiation temperature and sources, thus, the highest efficiency is difficult to be concluded for the Zn-diffused GaSb TPV cell technology. Recently, research efforts focus on optimizing the GaSb TPV cell structure to improve the cell performance. For example, the incorporation of wide bandgap window layers and optimization works on the cell configuration have been widely investigated.

In terms of GaSb TPV cells implementation, Khvostikov et al. [133] compared the performance of Zn-diffused GaSb-based TPV arrays connected in both series and parallel configuration. The parallel connection in the cylindrical system was found to produce a better performance when compared to the series connection in a conical system. A photocurrent of 3.58 A was recorded for cylindrical GaSb-based TPV arrays. Consecutively, Khvostikov further measured the performance of a cylindrical TPV system under tungsten (W) and silicon carbide (SiC) radiators [134]. A cell performance of $V_{oc}$ = 0.48, $J_{sc}$ = 4.5 mA/cm^2^ and FF = 65% was reported under a 1900 K SiC radiator temperature. Furthermore, a cell efficiency of 19% was recorded using a W radiator. The configuration of photoconverters in the TPV system was well-studied, where higher power output can be produced with higher number of photoconverters. On the other hand, Wu et al. [135] performed an experimental analysis on a TPV system with a SiC radiator to compare the output power density of a single GaSb and Si cell under the same operating condition. It was reported that the GaSb cell produces up to 11 times higher power density when compared to a single Si cell. An output power density of 0.26 W/cm^2^ was realized for a single GaSb-based TPV cell under an operating SiC radiator of 1223 K temperature.

### 4.3. Performance Improvements

Research efforts towards maximizing the conversion efficiency have been drastically increased in recent years. Improvements in terms of spectral control [137], metal contact [138,139,140,141,142] as well as optimization on the layer thickness [51,92,117] and doping concentrations [50,51,143,144] are the major topics reported from various works.

#### 4.3.1. Metal Contact Optimization

A metal contact is required to generate electricity by providing a connection between the active semiconductor devices and the external circuit [139]. Good metal contact with low electrical resistance, often referred to as an ohmic contact, is essential to minimize the loss of electrical power and maximize the overall efficiency. The properties of an ohmic contact include good thermal stability, low resistance and good adhesion on the semiconductor surface [139]. Milnes and Polyakov [145] discussed that formation of an ohmic contact on p-type GaSb may involve Au or AuZn for doping concentration of 6 × 10^−17^ cm^−3^. Consecutively, Milnes et al. [146] studied and compared Au, Ag and In for p-GaSb with doping concentration between 8 × 10^16^ cm^−3^ and 1 × 10^19^ cm^−3^. It was found that the contact resistivity is inversely proportional to the doping concentration. At doping concentration of 10^18^ cm^−3^, the contact resistance exhibited values around 5 × 10^−5^ Ωcm^2^. From their finding, it was demonstrated that lower contact resistances are favorable to form ohmic contact on p-GaSb.

On the other hand, materials such as nickel (Ni) and palladium (Pd) metals are found to be suitable candidates to form an ohmic contact on n-GaSb. In contrast to Pd metal, the intermetallic formation of Ni can be performed at a lower temperature. Rahimi et al. [139] reported that Ni metal can easily form a contact on n-GaSb and produces lower resistance and better ohmic characterization than a Pd metal. Other than that, Ge, gold (Au), platinum (Pt) and molybdenum (Mo) metals are often deposited to form a metallization scheme that produces good ohmic contact on the n-GaSb semiconductor. Each metal shows the advantage of low contact resistance towards the semiconductor. In particular, Rahimi et al. [141] reported a remarkable reduction in contact resistance by optimizing the annealing temperature windows of different metallization schemes for n-GaSb//(Pd+Mo)/Ge/Au/Pt/Au and n-GaSb//Ge/Au/(Pd+Mo)/Pt/Au metallization.

It is worth noting that metal contact is influenced by the doping concentration of semiconductor. Therefore, the difficulty to remove the oxide layer from a highly doped n-type GaSb material prior to the contact metallization is a disadvantage in the formation of ohmic contact. The fabrication and metallization techniques should be further developed to facilitate the formation of a good ohmic contact on n-GaSb, which supports the high photogeneration in TPV cell. Table 3 summarizes the reported metallization, specific contact resistance and metal-forming process on GaSb semiconductor layers.

#### 4.3.2. Thickness, Doping and Design Structure Optimization

The GaSb TPV cell performance parameters exhibit a strong dependence on the layer thickness, doping concentration and junction configuration. Therefore, researchers optimize these parameters to achieve maximum performance efficiency. An optimization of Zn-diffused GaSb emitter can improve the cell performance due to the formation of stronger built-in field that leads to a better collection of minority charge carriers [148]. Most studies reported that a thin layer of p-type emitter for the GaSb TPV cells is feasible to produce good quantum efficiency. Rajagopalan et al. [149] conducted an experimental study to obtain the optimum emitter depth of a GaSb TPV cell fabricated by a single-step diffusion method. The optimum junction depth that produces the maximum power output from the fabricated cell is around 0.4 µm. By decreasing the emitter depth, the quantum efficiency (*QE*) performance of the cell is increased while the $V_{oc}$ is decreased. On top of that, Shaimaa et al. [92] emphasized the trade-off relation between the emitter thickness and the performance parameter, especially with the $V_{oc}$ and $J_{sc}$ values. In this regard, a thick p-doped emitter with a high doping concentration shall therefore decreases the minority carrier lifetime hence decreases the $J_{sc}$ value. Contrarily, $V_{oc}$ is reduced by a thin emitter caused by the increasing leakage current of the cell. Additionally, Bett et al. [150] demonstrated a strong dependency on the quantum efficiency when the emitter thickness was decreased from 0.72 to 0.22 µm in the simulation work. Therefore, the necessity to develop detail optimization with the precise control of the emitter thickness is very crucial.

Recently, Tang et al. [50] performed a doping and depth optimization on a Zn-diffused GaSb cell for both emitter and base regions through simulation work. The doping of both regions was optimized to increase the *QE* in a range of long wavelengths. For an n-type GaSb substrate, increasing the doping concentration tends to reduce the power output of the cell. As for the p-type GaSb emitter layer, a moderate Zn concentration is sufficient to achieve high *QE*. Optimization on the GaSb structure has been done in recent years. In 2015, Tang et al. [51] performed a numerical simulation to investigate the GaSb cell designed to be an inverted “n on p” configuration through Tellurium diffusion into unintentionally p-doped GaSb TPV cell. The n on “p” configuration with a thin optimal diffusion n-emitter depth of 0.1 µm and a thick p-base shows better performance than the “p on n” GaSb TPV cell configuration. This is because a thick p-base generates a high amount of electron minority charge carriers. In addition, the carrier extraction is improved due to the long diffusion length. Therefore, a higher power density and the *IQE* of the cell were attained in [50,51,151]. Under 1500 K blackbody radiation, the *I_sc_* for “n on p” configuration was improved by a factor of 1.43 as compared to the “p on n” GaSb cell configuration.

With regards to the anti-reflective coating (ARC), Fraas et al. [136] discussed the design optimization incorporating an “n+” transparent conductive oxide (TCO) layer with a hydrogenated amorphous silicon interface (Si:H) passivation between the n+ and p-type GaSb base. The integration serves as a built-in plasma filter by reflecting the IR radiation at a longer wavelength back to the radiation source hence improving the overall performance. Recently, Tang et al. [152] demonstrated the n-type vapor diffusion by depositing a silicon oxide layer on the p-GaSb surface. In comparison to a traditional p-on-n GaSb TPV cell structure, higher *QEs* at longer photon wavelengths have been reported, resulting in 1.42 times higher output power density recorded under 1573 K temperature.

Additionally, optimization work has been done on the TPV filter by a research group from Tufts University, USA. Licht et al. [151] demonstrated a 10% improvement in *IQE* with optimized design of a GaSb TPV cell by incorporating a metallic photonic crystal as the front-surface filters (MPhCs). It is claimed that the repeating nanoscale structure of the MPhCs only allows the transmission of near bandgap radiation as this structure creates a photonic bandgap and provides a narrowband confinement of the incident photons. Additionally, the formation of an evanescent field penetrates the GaSb cell results in higher photon absorption. Therefore, MPhCs are coined to reduce various types of recombination mechanisms. Despite all of the advantages, Licht’s studies are mainly based on a simulation tool (Silvaco Atlas software), and cell fabrication based on the design structure is yet to be demonstrated and further explored.

## 5. InGaAs-Based TPV Cell

In this section, a comprehensive study on InGaAs-based TPV cell in terms of their background, cell performance, improvements and challenges are thoroughly discussed.

### 5.1. Introduction

InGaAs is a III-V ternary semiconductor compound of InAs and gallium arsenide (GaAs) that consists of indium and gallium group III elements, and arsenide group V element. The bandgap energy of InGaAs can be engineered from 1.42 to 0.36 eV by having a variation of *x* composition in In_x_Ga_1-x_As, corresponding to cut-off wavelength from 0.87 to 3.34 µm [4]. At *x* = 0.53, In_0.53_Ga_0.47_As is lattice-matched to indium phosphide (InP), corresponds to *E_g_* and *λ_c_* of 0.74 eV and 1.68 µm, respectively. On the other hand, InGaAs cell with a *E_g_* lower than 0.74 eV will have a longer *λ_c_*, this will improve the harvesting of mid-IR and hence increase the output power [153]. A lattice-mismatched InGaAs with higher indium composition is designed to arrive at a lower bandgap. InGaAs cells have the advantage of achieving *λ_c_* up to 2.6 µm. However, the mismatch between the InGaAs and InP substrate results in higher dark current.

In 1980s, Woolf [17] at GA Technologies (San Diego, CA, USA) was the first to propose InGaAs photoconverters for TPV applications. In the 1990s, the National Renewable Energy Laboratory, US (NREL) played a significant role in the collection and promulgation of TPV data, where TPV research on lattice-matched and lattice-mismatched InGaAs were introduced. Those studies involved the theoretical calculation, growth, fabrication and characterization of InGaAs TPV cells under solar and TPV spectrums. Recently, studies used an InGaAs cell in multi-junction such as GaInP/GaAs/InGaAs [103], and tandem cells such as GaAs/InGaAs [154], which aim to increase the total efficiency and to extend the power harvesting into the mid-infrared wavelengths. Several studies reported the crystal defects of lattice mismatch InGaAs cells for bandgap from 0.55 to 0.6 eV [155], characterization of the cell performances and optimization of the structure design [156,157,158,159].

### 5.2. Performance of InGaAs-Based TPV Cell

Typical lattice-matched In_0.53_Ga_0.47_As cell has *V_oc_*, *I_sc_*, *FF* and *η* ranging from 0.3 to 0.4 V, 21.5 to 57.7 mA/cm^2^, 66 to 74.2%, and 4.2 to 14.37%, respectively, under AM 0 and AM 1.5 illuminating condition [101,102,103]. On the other hand, lower bandgap lattice-mismatched InGaAs cells have recently received tremendous attention due to the diversified applications. The indium ratio was increased while the ratio of gallium was decreased to produce a lattice-mismatched InGaAs [160]. Reducing the bandgap of InGaAs will increase the *I_sc_* resulting in higher output power. On the other hand, there is a limitation to fabricate a high-performance lattice-mismatched cell which has a lower bandgap on InP substrate. InGaAs with lower bandgaps show a higher degree of Auger recombination, whereas excessive defects due to lattice-mismatched epitaxy cause a high level of Shockley-Read-Hall (SRH) recombination. Both Auger and SRH recombination will increase the dark current and limit the device performance [161]. Recently, Zhou et al. [162] investigated the utilization and optimization of TPV system cavity, which consists of emitter, PV cells, and mirrors to modify the spatial and spectral distribution within the system. It was demonstrated that careful design of the cavity configuration can significantly enhance the performance of the TPV cell.

Moving on, Tan et al. [159] compared the performance of In_0.53_Ga_0.47_As and In_0.68_Ga_0.32_As cells under blackbody temperatures from 800 to 1323 K. As the blackbody radiation temperature increases, the *λ_p_* of the radiation spectrum gradually shifts to the shorter wavelength. The amount of radiation photons that can be absorbed by the TPV cell considerably increases, leading to higher *J_sc_*, *V_oc_*, *FF* and *P_out_*. The In_0.53_Ga_0.47_As and In_0.68_Ga_0.32_As cells obtained an *η* of 16.4% and 19.1% at 1323 K blackbody temperature, respectively. The *η* continues to increase as the radiator temperature increases. However, the optimum *η* cannot be obtained due to the restriction of the blackbody source with a maximum heat temperature of 1323 K. InGaAs cells have shown different performance under TPV and solar spectrums. Cells tested at TPV spectrum have reported higher *J_sc_*, *V_oc_* and *P_max_* when compared to solar spectrum [159,163,164]. The increment in cell performance is related to the illumination intensity of the spectrum, which tends to be higher at TPV spectrum or concentrated solar spectrum [165,166]. On the other hand, lower *FF*, higher saturation of current density and ideality factor are reported when an InGaAs cell was tested under TPV radiation spectrum as compared to AM 1.5 spectrum. The change in the radiation spectrum has a significant effect on current mechanisms of InGaAs cell, which considerably dominates the minority carriers transport process. Recently, Gamel et al. [165] highlighted the effect of various spectral irradiances (different radiation temperature at different beam intensity) on the performance of TPV cell. The *J_sc_* increases linearly with illumination intensity while *V_oc_* increases logarithmically. Meanwhile, the *FF* is expected to reduce with increasing illumination intensity, especially if the physical structure design of the TPV cell does not support the collection of high photogenerated carriers. Such a phenomenon is related to the high resistance losses.

The performance of various single lattice-matched and mismatched InGaAs cells are presented in Table 4. Recently, the growth optimization of high crystal quality lattice-mismatched InGaAs TPV cell has gained considerable attention [156]. The buffer layer plays a key role in developing high-quality lattice-mismatched InGaAs TPV cells. The effect of different buffer structures, such as a single buffer layer, compositionally graded buffer layers and superlattice buffer layers, were investigated [156,167,168,169]. A lattice mismatch of ∼1.2% was introduced between In_0.68_Ga_0.32_As and a compositionally nonmonotonically graded InAsP buffer layer (14 layer grades) [155,156]. On the other hand, Hudait et al. [85,91] has grown In_0.69_Ga_0.31_As using the MBE method and reported a 1.1% lattice-mismatch between the device layer and the InP substrate. Recent work by Lian et al. [156] also grew In_0.69_Ga_0.31_As using MOCVD method. Under the AM 1.5 spectrum, the *J_sc_* and *V_oc_* are 47.6 mA/cm^2^ and 0.35 V, while the *η* is 6.9%.

Aside from that, Zhang et al. [177] reported a *λ_c_* at 2.5 μm for In_0.8_Ga_0.2_As material, the large lattice-mismatched between the epilayer and substrate cause defect, which affects the quality of the material in both electrical and optical properties. The lattice-mismatched problem was solved using two-step growth methods. Firstly, the thin buffer layer was grown with low temperature. Next, the epilayer was grown with a higher temperature. This approach avoids propagation dislocation, thus improves the crystalline quality of epilayer [91,167,168,169,177,178]. In summary, extended InGaAs have received attention in photodetector and sensor application, but there is limited work on the characterization of extended InGaAs for TPV application. Therefore, the optimization and characterization of extended InGaAs TPV cells should be systematically carried out to fully exploit their potential in TPV application [177,179].

### 5.3. Performance Improvements

Research effort towards optimizing the cell performance has drastically increased in the recent years. Optimization on the layer thickness, doping concentrations, as well as Monolithic Interconnected Modules (MIMs) are the major topics reported from various works.

#### 5.3.1. Metal Contact Optimization

Photogenerated carriers in the semiconductor are transferred through the front and back metal contacts, one metal exchanging electrons and the other exchanging holes. For ohmic contact resistance, the n-conductor work function must be much greater than that of the semiconductor, and for a p-conductor much smaller than that of the semiconductor. Under AM 1.5 testing condition, n-p and p-n InGaAs have similar performance due to the long minority carrier diffusion length and good surface passivation [52,164]. However, larger contact resistance in the p-n configuration required a greater surface grid coverage to avoid *FF* loss, leading to much higher shading loss and a significant reduction in efficiency per total area (7.2%) [164].

Ohmic contact formation on InGaAs TPV cell is similar to GaAs PV cell, where the ohmic contact is usually prepared with e-beam evaporation of a metallic multilayer structure in one pump down cycle. For n-InGaAs, the ohmic contact resistance decreases from $10^{-6}$ down to$\;10^{-8}$ ${\Omega \mathrm{cm}}^{2}$, when the dopant increases from $10^{16}$ to $10^{19}{\mathrm{cm}}^{-3}$. The n-InGaAs has a specific contact resistance $\rho_{c}=10^{-7}-10^{-8}\;{\Omega \mathrm{cm}}^{2}\;$[180]. For p-InGaAs, higher contact resistance can be achieved as compared to n-InGaAs. The vast majority of lattice-matched and mismatched InGaAs TPV structure reported the use of AuGe and Ti/Au as front metal contact and Au and TiAu as back metal contact [85,102,164] to produce an ohmic contact. Tan et al. [155] used Ni/AuGe/Ni/Au metallization scheme for n-type ohmic contact, and Pd/Zn/Pd/Au metallization was used for p-type ohmic contact. Recently, Kao et al. [132] reported the utilization of AuGe/Au metallization for n-type InGaAs layer. It can be ohmic contact with n+-GaAs and n+-InGaAs contact layers without thermal annealing due to the heavy doping of these layers. Ohmic contact formation between the p-InP substrate and the AuBe/Au metal electrode is an essential factor to improve *η* up 14.37% by receding the series resistance. Recently, Li et al. [157] proposed Tb/Ni/TiN metal stack for n-InGaAs with lower specific *ρ_c_* of 7.98 × 10^−9^ Ω cm^2^. Increasing the dopant concentration in the Ni-InGaAs alloy and the lowering the barrier height between Ni-InGaAs and n-In_0.53_Ga_0.47_As successfully increase the contact resistance.

#### 5.3.2. Thickness, Doping and Design Structure Optimization

The variation of layers’ thickness and doping concentration directly affect the output performance of InGaAs TPV cell. For example, the increment of absorber thickness will increase the $J_{sc}$ as more photons are absorbed. On the other hand, $V_{oc}$ decreases with the available length over which carriers’ recombination can occur. Moreover, doping density will significantly affect the lifetime and mobility of carriers. Finding the optimum thickness and doping concentration of InGaAs TPV cell will maximize its output performance [102,103].

Most of the literature reported a very thin layer of emitter layer from 0.1 to 0.3 µm [155]. Thinner highly doped emitter is able to produce lower resistance depending on the metal contact and able to generate a feasible electric field with the base layer. Aydin et al. [158] reported an optimum thickness (doping concentration) of 0.5 µm (1 × 10^17^ cm^−3^) for emitter layer and 3.5 (1 × 10^17^ cm^−3^) for a base layer under 1400 K blackbody radiation. Emrnzian et al. [171] used PClD software to optimize the thickness and doping concentration of the emitter and base layers of In_0.53_Ga_0.47_As under 0.5 W/cm^2^ at 3300 K blackbody temperature. Optimum cell performance is reported at base and emitter thickness of 2 µm and ~0.2 µm. In [170], the doping concentrations and thicknesses of the emitter and base layers of In_0.53_Ga_0.47_As TPV cell were optimized under 2000 K and 5000 K blackbody temperatures. It was reported that *J_sc_* and *η* steadily improve as the base thickness increases from 0.5 to 2 µm, but the increasing trend saturates as thickness further increases beyond 2 µm. Emitter thickness <0.3 µm and doping concentration of ~1 × 10^17^ cm^−3^ provide optimum device performance. Under 2000 K and 5000 K blackbody temperature, 0.3 µm and 3 × 10^17^ cm^−3^ for the emitter, and 2 µm and 1 × 10^17^ cm^−3^ for the base were reported [170]. Recently, Gamel et al. [181] reported a multi-dimensional optimization of In_0.53_Ga_0.47_As TPV structure using the real coded genetic algorithm at radiation temperatures between 800 to 2000 K. Cell efficiency increased by an average percentage of 11.86% as compared to the non-optimized cell. It was highlighted that the integration of a thicker base layer with the back-barrier layers enhance the generation of charge carriers and increases the collection of photogenerated carriers near the band-edge.

The majority of InGaAs devices are epitaxial grown heterojunction. The active junction is combined with cap, window, buffer layers, which play an important role in improving device performance. A very thin and heavily doped InGaAs cap layer <1 × 10^19^ cm^−3^ obtains a low resistance (Ohmic contact) with the front metal contact and selectively removed except at the contacts to avoid optical absorption by the cap layer [102,166]. Jain. [182] investigated the effect of p-InP window layers for lattice-matched In_0.53_Ga_0.47_As cells using PC1D software. The *EQE* results demonstrated the suitability of window layer to reduce front surface recombination resulting in a significant improvement. The large conduction band discontinuity at the window/emitter interface provides a tremendous potential barrier for the movements minority carriers and reduces the surface recombination of electrons. It was found that under AM 1.5, devices with 1.5 µm window layer produce lower *J_sc_* as the majority of light spectrum is absorbed by InP layer. For TPV application, significant portion radiated energy will be lower than *E_g_* of InP. The *R_s_* value can be further minimized by optimizing the physical parameters (doping and thickness) of the entire structure. Ming et al. [155] presented an improvement in TPV device for an In_0.68_Ga_0.32_As cell with optimized material growth, suitable absorber thickness of 2.8 µm as well as TiO_2_/SiO_2_ ARC with 2.1 µm InAsP buffer layer. Such enhancement has increased the $V_{oc}$ from 0.19 to 0.23 V, the $J_{sc}$ from 43 to 56 mA/cm^2^, and the conversion efficiency from 5.31 to 8.06% under AM 1.5 test condition. Research focuses on single-variable optimization to improve on the TPV cell *η,* the research gap that worth to be highlighted is to perform multi-variables optimization to achieve a higher cell *η.*

#### 5.3.3. Advancement and Improvement in InGaAs TPV Cell

For TPV cells improvement-wise, optimizing the ARC materials and thickness play a key role in decreasing the reflection at the front surface of the InGaAs cell. Other methods like the rear mirror reflector, Lambertian rear reflector and textured surface are used to improve the light absorption and light trapping in the cell [183]. Jurczak et al. [184] investigated the effect of ambient temperature and light trapping (rear reflector and textured surface) on the performance of InGaAs using Matlab. Results show a 3–5% improvement when the light trapping is implemented. Recently, Burger et al. [185] suggested the potential for a dramatic increase in conversion efficiency through improved spectral selectivity and recycling of longer IR, combined with the potential for reduced module costs through wafer reuse using thin-film TPV. The air-bridge InGaAs thin-film device with gold rear mirror reflector was investigated to realize highly selective absorption of above-bandgap radiations, enabling more efficient photon recycle and improving the TPV performance. Thin-film exhibits higher average sub-bandgap reflectance of 99% comparing to <95% sub-bandgap reflectance of conventional cell. The simulation suggests a TPV cell exhibiting these properties operates with an efficiency above 30% with 1455 K SiC radiator. Other method suggested the use of a closed system chamber which combined the radiator and InGaAs with near mirror reflector. The illumination reflected from the rear reflector recycled back in the radiator creating light trapping TPV system. Such improvement makes it possible to achieve system efficiency >50% [186].

Monolithic interconnected modules were implemented for the TPV system to improve the system efficiency [187]. MIM provides several advantages, firstly is that series-connected cells generate high voltage and low current, the array size can be designed to minimize the loss due to non-uniform radiator temperature. Also, the MIM cell can be directly connected to the substrate or heat sink without any electrical isolation limitation concern [188]. The lattice-matched InGaAs/InP MIM of 0.74 eV bandgap energy and lattice-mismatched MIM of 0.55 eV bandgap were studied [189]. Fifteen InGaAs cells in series connection with an active area of 0.9 cm × 1 cm were tested, and a gold BSR was recommended for efficient radiation recuperation. The literature shows that InGaAs MIM TPV cell with 0.74 eV recorded $V_{oc}$ of 6.16 V, FF of 0.74 and $J_{sc}$ of 0.84 A/cm^2^, while InGaAs MIM TPV with 0.55 eV recorded $V_{oc}$ of 4.85 V, FF of 0.58, and $J_{sc}$ of 3.87 A/cm^2^ [189]. This study shows that series connected MIMs generate high voltage and low current. The reliability of MIMs could be further improved by designing series-parallel string MIMs. Moving on, Wehrer et al. [190] further explored the TPV tandem converter technology to improve TPV efficiency while sustaining high power densities. The study was conducted on an epitaxial grown lattice-matched In_0.53_Ga_0.47_As with 0.74 eV bandgap energy and lattice-mismatched In_0.64_Ga_0.36_As with 0.63 eV bandgap energy. Under a 1273 K of blackbody source radiation and 325 K of cell temperature, $V_{oc}$ of 6.14 V, *I_sc_* of 0.292 A/cm^2^, and FF of 0.676 were attained [190].

Other improvements are presented by matching the radiation spectrum to the spectrum response of InGaAs devices. Woolf et al. [191] reported 43% and 52% of spectral efficiency at 1300 K and 1500 K for In_0.68_Ga_0.32_As TPV cell with cell efficiency of 22% and 26%. In 2017, David et al. [17] presented the novel selective thermal radiator to improve the efficiency of TPV system with source temperature above 1000 °C and a In_0.68_Ga_0.32_As TPV cell. Using the conventional stepper lithography, the radiator was produced using a passivated platinum and alumina frequency selective surface. A power conversion efficiency of 24.1% was achieved at 1055 °C of radiation temperature with spectral efficiency of 80%. In 2018, Fekadu et al. [48] numerically analyzed a multilayer optical radiator based on metamaterial design which exhibited polarization and azimuthal angle independent selective emission for In_0.68_Ga_0.32_As TPV cell. The proposed multilayer TPV cells achieved an enhanced broadband and a wide-angle average emissivity of 96.6% between 0.2 to 2.1 μm. Spectral efficiency of 79.6% was achieved for TPV cell at *λ_c_* of 2.1 µm under 1400 K source energy as compared to 56.6% for a single layer. Recent improvement in the TPV cells and system improve the conversion efficiency and output power, hence increases the feasibility and practicality of TPV in various terrestrial and space applications.

## 6. Narrow Bandgap Materials for TPV

TPV cells utilize narrow bandgap materials which allow them to harvest the maximum amount of infrared radiation. Aside from the available TPV materials: GaSb TPV cell discussed in Section 4 and InGaAs TPV cells in Section 5, there are several narrow bandgap materials that worth highlighting. Single element semiconductor like Ge with a bandgap of 0.66 eV, binaries like InAs with a bandgap of 0.35 eV and indium antimonide (InSb) with a bandgap of 0.17 eV and ternary like indium gallium antimonide (InGaSb) with a bandgap energy range of 0.17–0.72 eV and indium arsenide antimonide (InAsSb) with a bandgap energy range of 0.1–0.4 eV and quaternary like InGaAsSb with a bandgap energy range of 0.5–0.55 eV and InAsSbP with a bandgap energy range of 0.3–0.5 eV are accessible narrow bandgap materials. There are several types of potential TPV materials as listed in Table 5, yet with some limitations and room for improvement in terms of TPV cell optimization and light trapping. These materials can be further explored for the potential to harvest waste heat temperature below 1200 K.

**Figure 3:**
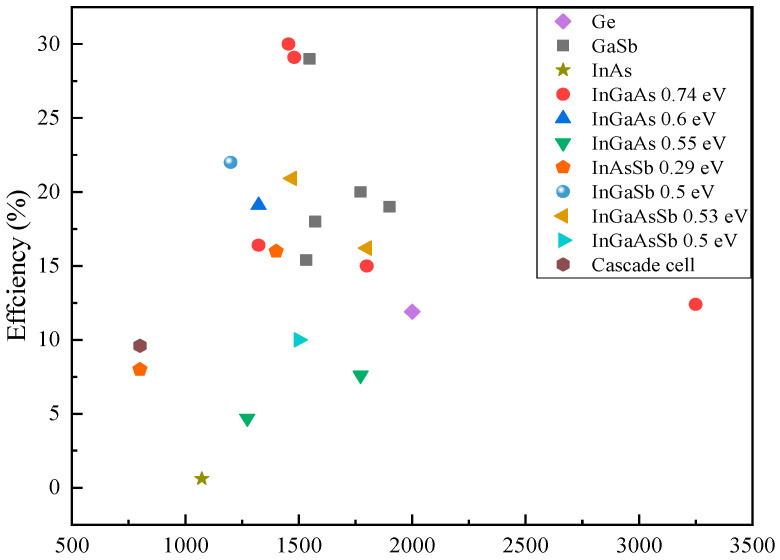
Record TPV cell efficiencies for various materials Ge [195], GaSb [3,131,134], InAs [204], InGaAs [52,103,159,163,185,186], InAsSb [205], InGaSb [200], InGaAsSb [63,198,199] and cascade InAs/GaSb/AlSb [201].

Theoretically, TPV cells made by narrower bandgap semiconductors are able to absorb photons up to longer wavelengths, resulting in better cell performance. However, there are several drawbacks of narrow bandgap (<0.7 eV) TPV cells such as low *V_oc_*, high dark current, and immature growth technology, and nonoptimal structure design. Recently, Gamel et al. [192] compared the performance of various reported narrow bandgap cells under 1000 K blackbody temperature. Based on their simulation result, In_0.53_Ga_0.47_As has better *IV* curve characteristic in comparison to Ge, GaSb and InAs, due to the mature structure with FSF and BSF layers which results in lower surface recombination. InAs with the long $\lambda_{c}$ of 3.5 µm produced an output power of 36.7 mW/cm^2^ as compared to 42.1, 27.7 and 6.3 mW/cm^2^ produced by In_0.53_Ga_0.47_As, GaSb and Ge cell, respectively. Furthermore, InGaAsSb is more promising as compared to GaSb and Ge, due to the high cell efficiency and its ability to operate in the low range blackbody temperature. Furthermore, Table 5 and Figure 3 summarize and compare the characteristics of narrow bandgap materials for TPV cells. Based on the comparison in Table 5, aside from extended InGaAs, InGaAsSb has the best potential among the narrow bandgap materials for radiation low-temperature (<1273 K) due to its promising $V_{oc}$ and $J_{sc}$, yet further cell optimization is needed to improve the cell efficiency. Based on the current literature, InAsSbP TPV cell has limitation in achieving a promising *FF* and $V_{oc}$, with epitaxial and diffusion emitter, the$\;V_{oc}$ recorded at 0.1 V [193]. InGaAsSb and InSb are therefore worth to be further optimized and improved for TPV application. Furthermore, as illustrated in Figure 3, InGaAs (0.74 eV) and GaSb TPV cells recorded the highest cell efficiencies, which demonstrates the maturity of these structures.

**Table 5:** Narrow Bandgap Materials TPV cells.

Ref	Material	T_rad_	T_cell_ (K)	$\lambda_{c}$ (μm)	$E_{G}$ (eV)	V_oc_ (V)	I_sc_ (A)	$J_{sc}$ (A/cm^2^)	FF (%)	$\eta_{cell}$ (%)	Exp./ Sim.
[194]	Ge	AM 0	300	1.9	0.66	n/a	n/a	n/a	n/a	13	Exp
[195]	Ge	2000 K (3.66–36.6 W/cm^2^)	300	n/a	0.664	0.323–0.383	5 × 10^−7^–5.1 × 10^−6^	n/a	73.4–76.4	9.6–11.9	Sim
[196]	Ge	AM1.5 × 25 sun	298	~1.6	0.6	0.34	0.89	n/a	68	28.8	Exp
[197]	In_0.2_Ga_0.8_Sb	AM 1.0	N/A	2.2	0.56	0.24	n/a	0.037	n/a	34.33	Sim
[198]	In_0.2_Ga_0.8_As_0.17_Sb_0.83_	1800 K	300	2.4	0.53	0.35	n/a	36.3	n/a	16.2	Sim
[63]	In_0.15_Ga_0.85_As_0.14_Sb_0.86_	1470 K	300	2.4	0.53	0.327	n/a	3.01	65	20.92	Exp
[59]	InGaAsSb	1100 K	N/A	~2.4	0.53	-.18		0.5	n/a	n/a	Exp
[199]	In_0.16_Ga_0.84_As_0.14_Sb_0.86_	1500 K	300	2.44	0.506	0.3–0.5	n/a	50	65	10	Sim
[200]	In_0.25_Ga_0.75_Sb	1200K	300	2.5	0.50	0.34	n/a	3.2	n/a	22	Sim
[62]	In_0.22_Ga_0.78_As_0.2_Sb_0.8_	1328 K	300	~2.5	0.49	0.187–0.231	n/a	1.042–2.170	44.2–46.9	n/a	Exp
[62]	In_0.22_Ga_0.78_As_0.2_Sb_0.8_	1328 K	300	~2.52	0.49	0.208–0.240	n/a	6.895–8.815	51.3–54.9	n/a	Sim
[78]	Ga_0.78_In_0.22_As_0.20_Sb_0.80_	AM 1.5 and 1073 K	N/A	~2.52	0.50	0.074 and 0178	n/a	0.035 and 0.98	33 and 46	n/a	Exp
[201]	Cascade InAs/GaSb/AlSb	800 K	300	3.1	0.40	0.799	17.9 m	43.5	51.4	9.6	Exp
[202]	InAsSbP	AM 1.5	N/A	3.5	0.39	0.12	n/a	3	n/a	n/a	Sim
[203]	InAs	1223 K	300	3.5	0.35	0.06	n/a	0.9	n/a	n/a	Exp
[204]	InAs	1073 K	300	n/a	0.36	0.027	n/a	0.267	27	0.61	Sim
[205]	InAs_0.91_Sb_0.09_	800–1400 K	300	4.33	0.286	0.83–0.154	n/a	1.9–30	n/a	8–16	Sim
[206]	InSb	773 K	300	n/a	0.235	n/a	n/a	n/a	n/a	n/a	Sim
[207]	InSb	1273 K	300	n/a	0.235	0.085	10 × 10^−6^	n/a	64	n/a	Exp

## 7. TPV Applications

**Figure 4:**
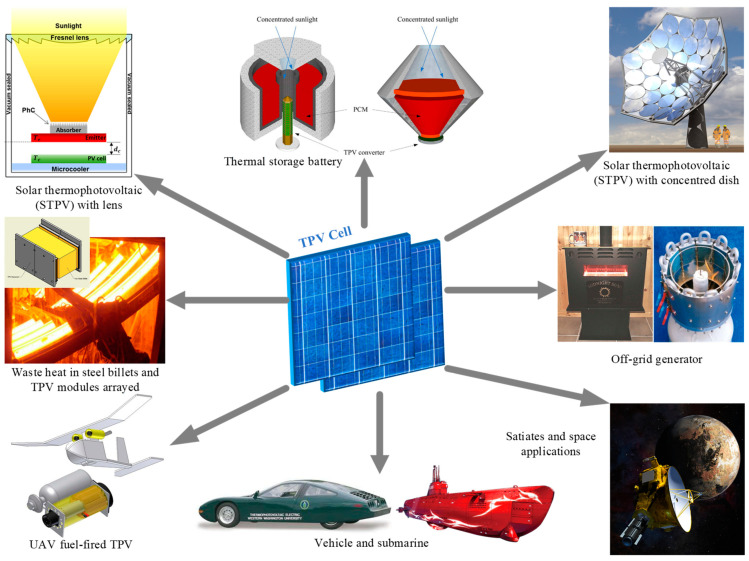
TPV applications.

TPV system received tremendous attention due to its promising contribution as economical, efficient, practicable power systems, and clean power generation. The application of TPV can be categorized based on the chemical reaction or nuclear fusion reaction types of thermal heat source. These heat sources are categorized into solar heat, combustion of fuels, nuclear sources and waste heat. Figure 4 presents the application for TPV technology.

### 7.1. Solar TPV Systems (STPV)

In principle, STPV system utilizes the solar energy to heat up the radiator to <3273 K through a solar concentrator. These high-temperature radiators will then emit thermal radiation to the TPV cells that converts the infrared photons into electricity [208]. STPV work has been reported on Fresnel point-focus and dish concentrators, which recorded a temperature up to 1623 K [4]. Xuan et al. [208] emphasized that the configuration of radiators influences the distribution effect of radiator temperature. A cylindrical radiator was found to give the most stable performance for STPV system. Zhou and associates [209] performed a comprehensive review of TPV cells material in STPV system. In contrast to Si and Ge cell, GaSb and InGaAs TPV cells show better performance in STPV system. Nevertheless, the cost of material and fabrication process remain a big challenge. Furthermore, a multi-bandgap cell such as GaInP/GaAs/Ge shows the potential of integration into a STPV system, which possesses higher conversion efficiency as compared to a single junction cell [4]. Nowadays, the STPV system in a hybrid system attracts considerable interest. Hussain et al. [210] conducted a performance analysis on different arrangement of hybrid STPV design using solar-biomass/gas power generation system. The hybrid STPV system can be operated at temperature lower than 1273 K, which widens the opportunity for other applications such as portable battery charger and microgenerator for household application. In addition, STPV hybrid with solar-natural gas has been demonstrated to give an electrical power output around 500 W and a conversion efficiency of 22% [211]. This architecture offers a promising development in the near future.

### 7.2. Combustion-Driven TPV Generators

Combustion-driven TPV system can be widely considered for micro-, meso-, and macro-scale power supply applications [212]. Practical applications of combustion-driven TPV generator include portable electric generator, combined heat and power (CHP) as well as hybrid electrical vehicles. Portable TPV system was highly recognized in military application, aiming to replace heavy batteries and noisy diesel-electric generators [2]. Collaboratively, JX Crystal Inc. developed the first hydrocarbon-fueled TPV generators using GaSb TPV cell known as “Midnight Sun”, producing electrical power up to 100 W [121]. McDermott Technology Inc (MTI) has then teamed up with JX Crystal and developed a 500 W diesel-fueled portable TPV power generator. In addition, Chan et al. [213] developed a portable microgenerator with the combination of propane-fueled TPV microgenerator, photonic crystal radiator, and low bandgap InGaAs-based TPV cells. After a thorough system analysis, Chan et al. [214] redesigned the micro burner and developed vacuum packaging to prevent convective loss to the photonic crystal. Results suggested that further development can be promoted for higher conversion efficiency. For home-scale operation, this technology is often called the off-grid generator where the system aims to provide heat and electricity to remote houses during the winter and night time.

The CHP-based TPV has not only been realized to provide good performance for home use, but the system is also applicable in larger scale such as the central heating furnaces in large buildings and industrial furnaces [215]. Limitation of TPV system in CHP is the low-grade heat generation due to heat dissipation during the cooling of TPV cells. The typical cell temperature in CHP mode is around 333 K, which has been suggested with an additional heat exchanger to upgrade the heat for the exhaust gas [4]. Exploitation of micro-CHP has been investigated on domestic boilers in residential area to provide central heating which converts waste heat into electricity [216]. Bianchi et al. [217] investigated CHP TPV for residential buildings, considering both the energetic and economical point of view. In addition, low bandgap tandem cell such as InGaAsP/InGaAs shows the potential of incorporation in CHP systems. In comparison to thermodynamic limit, a significant improvement was made for lower bandgap materials on the TPV cell conversion efficiency [218]. De Pascale et al. [219] introduced a thermodynamic analysis of CHP by integrating a TPV generator into an Organic Rankine Cycle. A 56% thermal efficiency and 24% electrical efficiency were achieved in optimum electrical load configuration.

In hybrid electric vehicle (HEV), the exhaust heat from gas-powered engine is converted by a TPV device to charge the batteries with sufficient power to accelerate and maintain a cruise speed. TPV generators which produce a power range from 6 to 10 kW were investigated for HEV [122,123]. The major concern for TPV generators in HEV is the requirement of high efficiency. For instance, a 10 kW output capability is needed to maintain a steady-state cruising at 113 km/h without drawing any power from the battery [122]. The first TPV-powered automobile prototype was built in 1999 and named as “Viking 29” [123], which utilized GaSb-based TPV cells. The TPV generator consists of 20 GaSb-based TPV cells connected in series and *V_DC_* of 126 V [122]. The challenge of low conversion efficiency for the currently available TPV systems would be more reliable to support a small vehicle with smaller power range [2]. Research efforts have been devoted towards future development for better heat recovery and improvement of the system efficiency to achieve high-performance HEVs. The design system with selective emitter and quantum well TPV cell theoretically capable of yielding 24.5% of efficiency and 6 kW of electric power to recharge the battery pack of an electric city-car. However, further experimental investigations are needed for the emitter-cell system [26].

### 7.3. Space Applications

Moving forward, high-efficiency TPV cells are essential in space technology application [4]. There are two feasible power sources to power up a small spacecraft for long duration, which are solar and nuclear generators [220]. Recently, a critical review was conducted on space power generation to compare the main competing technologies [10]. TPV system promises up to 40% of efficiency and additional advantages of lightweight, mechanically static, and direct electricity production from radiant heat in space power generation. Also, the TPV system generates high power density per unit area as compared to other technologies, which is suitable for medium electrical system. The power density can be further increased with the use of radioisotope TPV generators with the use of nuclear fission sources [220,221]. For nuclear generators, the heat range is typically from 10 kW to MWs [222], and these generators are currently under consideration for power generation in future planetary settlement missions [10]. The most recent development of RTPV has been made in the Institute for Soldier Nanotechnologies [223,224] where simulation and measurement results have been experimentally reported on RTPV prototype system using photonic crystal spectral control in terrestrial application. Moreover, a RTPV system designed with InGaAsSb cell was reported with 8.26% efficiency and output power of ~40 W [225,226]. A 0.6 eV InGaAs TPV cell is very useful in the space application with less than 1% of cell degradation in performance over years caused by the damaging effect in the system. The Plutonium-238 radioisotope was investigated with up to 3 W power output and 10% system efficiency at the Los Alamos National Lab [227].

### 7.4. Waste Heat Recovery (WHR)

Effort has been made by researchers and private companies in exploring the possibility of utilizing TPVs for power generation [228,229,230]. In the UK industrial activities, the potential of TPV system to recover waste heat was realized in high-temperature industry [228]. Turkish industrial sectors [125,230] estimated that the potential energy recovery using TPV system is around 22.40 to 67.45 PJ/year. Moreover, a recent thermodynamic analysis of TPV system for industrial WHR application reported that up to 7.31% of annual waste energy can be recovered [15]. Despite the great promise of a TPV system in the WHR application, low power conversion efficiency at waste heat temperature less than 1273 K has been a major concern [229]. This can be solved by implementing TPV cells with material bandgap lower than 0.7 eV such as InGaAs and InAs. Furthermore, the potential of TPV heat recovery in glass and steel manufacturing industry have been an attractive research area nowadays. In glass manufacturing industry, the TPV system is estimated to produce 270 kW power output from high-temperature object with a surface area of 27 m^2^ [231]. In addition, Fraas et al. [3] successfully demonstrated a TPV cell with power density output of 1.5 W/cm^2^ per cell generated from a hot glowing radiant tube burner (1548 K) at a steel industry. Besides, the potential of worldwide electricity production is estimated with the possibility to reach over 3.1 GW with TPV heat recovery system in steel industry.

### 7.5. Thermal Energy Storage System

TPV devices are of particular interest for thermal energy storage application. Conceptually, thermal energy storage system utilizes ultra-high temperature phase change materials (PCM). In this system, the energy is stored in the form of latent heat and transformed to electricity upon demand via TPV cells. Thermal storage system enables an enormous thermal energy storage density of ~1 MWh/m^3^, which is 10–20 times higher than that of lead-acid batteries, 2–6 times higher than Li-ion batteries as reported by Datas et al. [232]. Seyf and Henry [233] modelled a thermal energy storage system and identified the significant design parameters that affect the overall power cycle system efficiency. It was found that building systems at sufficiently large scales, integrating an effective BSR, increasing the *EQE* for photons above the bandgap will improve the system performance. Following that, the multi-junction or multiple TPV cells arranged optically in series can reduce the thermalization losses and may have the potential to exceed the efficiencies of combined cycles (~60%). In 2019, Amy et al. [234] employed ultra-high temperatures and multi-junction photovoltaics (2-junction cells) for thermal energy storage system. This new approach has several benefits including the ability to reach >50% roundtrip efficiency with a cost per unit power < $0.5 per W-e, and the potential to offer load following capabilities to grid operators.

## 8. Challenges and Recommendation

The market penetration of TPV system remains inconclusive due to the great challenges encountered throughout its technological development. In the next ten years, TPV technology is expected to become stable and technologically matured such that it is able to produce high power density output for future electricity generation. In this section, the issues and challenges in TPV technology on the overall TPV system, GaSb-based and InGaAs-based TPV cells are extensively discussed, together with the recommendation to overcome the challenges.

### 8.1. Spectral Mismatch to the Bandgap of TPV Cell Material

In principle, TPV cells operate at the optimum efficiency when the semiconductor energy bandgap is spectrally matched to the blackbody spectrum generated by the heat source [4]. Therefore, TPV cells material shall be chosen based on the bandgap, which correspond to the spectrum. This is to minimize optical loss cause by the spectral mismatch and poor absorption of photons by the TPV cell.

Tan et al. [159] compared the performance of In_0.53_Ga_0.47_As (0.74 eV) and In_0.68_Ga_0.32_As (0.6 eV) under various range of blackbody temperatures. It was found that the efficiencies of both cells gradually increased from 800 to 1323 K. The key reason for the efficiency increment is due to the positioning between cut-off wavelength of the materials and the peak emissivity ($\lambda_{p}$) of each temperature. As the temperature increases from 800 to 1323 K, the $\lambda_{p}$ shifts to a longer wavelength. At 1323 K, In_0.68_Ga_0.32_As records 3.3% higher efficiency as compared to In_0.53_Ga_0.47_As. The reason is because In_0.68_Ga_0.32_As (2.10 µm) has a nearer *λ_c_* with $\lambda_{p}$ at 1323 K (2.19 µm), in comparison to In_0.53_Ga_0.47_As (1.68 µm). The conversion of electricity effectively occurs at photon wavelengths near the *λ_c_* of a particular material. Assuming the highest photon-electricity conversion is achieved when *λ_c_* is approximately similar to the $\lambda_{p}$, the suitable operating temperature as a function of bandgap energy can be expressed by Equation (5).

(5) $$\lambda_{c}\approx \lambda_{p}\;\frac{hc}{E_{g}}\approx \frac{2900}{T_{BB}}\;T_{BB}\approx \frac{2900}{1.240}*E_{g}$$

**Figure 5:**
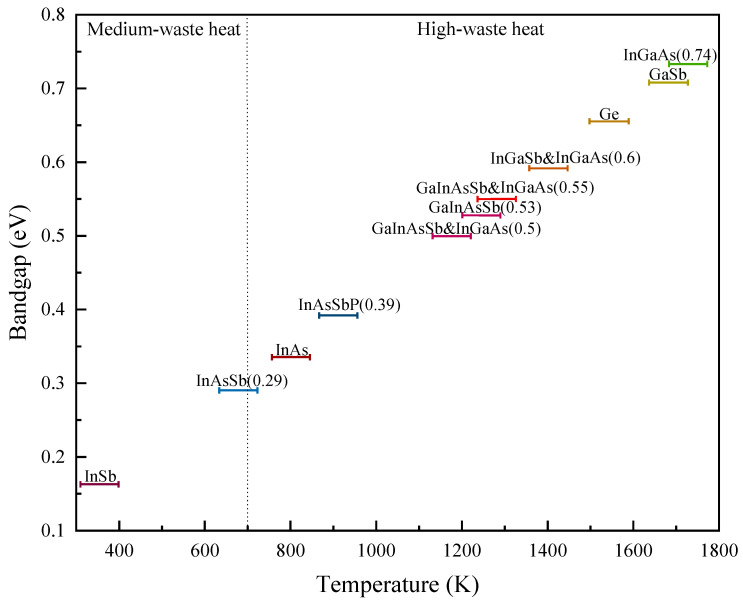
TPV cells and the blackbody temperature range of their optimum performance.

In this study, the suitable operating temperature for a particular material is suggested to be ± 10% of the *λ_p_*, as illustrated in Figure 5. For instance, the suitable temperature for InSb computed using equation 5 is approximately 397.58 K, which equivalent to $\lambda_{p}$ = 7.294 µm. A ± 10% of the *λ_p_* will give 6.5646 µm $\leq \lambda_{p}\leq \;$8.0234 µm, which corresponds to 361 K $\leq T_{BB}\leq$ 442 K.

Figure 5 shows that higher source of temperature is desirable for material with a higher bandgap energy. While InSb ($E_{g}$ = 0.17 eV) operates under lower range of blackbody temperature from 361 to 442 K, a lattice-matched InGaAs TPV cell ($E_{g}$ = 0.74 eV) operates at a blackbody temperature of 1600 to 1850 K. Furthermore, TPV cell can be matched with the emissivity of the practical radiator. Selective radiators can be engineered to emit at setting range of IR. In short, mismatching between the heat source spectrum and bandgap of the cell is one of the considerable issues that usually causes lower output energy and reduces the efficiency of TPV system. Therefore, the matching of blackbody spectrum to the suitable range of TPV cell bandgap energy is essential to generate the optimum amount of energy per unit area. The reflection and recycling of sub-bandgap photons in the radiator will significantly enhance the conversation efficiency of the TPV system.

### 8.2. The Effect of Cell Temperature

The TPV cells typically operate at temperature higher than 300 K and is located near to the heat radiator to achieve maximum light intensity. Since TPV cell is exposed to constant thermal radiation during its operation, the cell may heat up to a certain degree that may directly affect the cell performance [235]. Nevertheless, the increment of TPV cell temperature increases the $J_{sc}$ due to the reduction in the bandgap energy of semiconductor material. The decrease in $E_{g}$ allows more photons at slightly longer wavelength to be absorbed and create additional electron-hole pairs. The Varshni’s semi-empirical relation describes the effect of temperature on the bandgap energy as expressed in Equation (6) [236].

(6) $$E_{g}\left(T\right)=E_{g0}\;-\frac{aT_{cell}{}^{2}}{T_{cell}+b}$$

where *E_g_* *(T)* is the engineered bandgap at the setting cell temperature, $T_{cell}$ is the cell temperature, *E_go_* is the initial bandgap at room temperature, *b* is the room temperature in *Kelvin*, and *a* is the temperature coefficient.

However, the $V_{oc}$ decreases with an increase in cell temperature [237]. For example, Martin and Algora [235] reported that a GaSb TPV cell has an absolute temperature coefficient for $V_{oc}$ of −1.59 mV °C^−1^. Another factor of the decrease in $V_{oc}$ is that the dependence of the dark saturation current on the intrinsic concentration ($n_{i}$). The $n_{i}$ changes due to the $E_{g}$ dependence on the temperature. Therefore, when the $E_{g}$ reduces as temperature increases, higher $n_{i}$ will lead to higher dark saturation current, resulting in the lower $V_{oc}$.

The challenge of increased internal temperature of the TPV cells can be addressed by several methods. Apart from the incorporation of radiator and filter to protect the cell from thermalization effect, active cooling systems such as heat sinks, cycling coolant and forced-air coolant are commonly employed in numerous TPV prototypes [238]. Wu et al. [41] experimentally illustrated the integration of water-cooled mechanism in TPV system. The cooling system was operated using tap water, water flows through a series of parallel channels. On the other hand, coating the bottom of aluminum chassis heat sink with an additional thermal radiation layer has been demonstrated to enhance thermal radiation in concentrated solar cell by reducing 10 °C of the cell temperature [239]. A setup of cooling system for TPV applications using low-iron soda-lime glass was studied with opto-electro-thermal coupled simulation by Zhou et al. [240] to investigate the effect of temperature under different condition for the TPV application. Subsequently, further experimental work is required to examine this system in practical condition. The integration of TPV with PCM such as paraffin wax, graphene, nano-PCM [241] will lead to an effective cooling and thermal energy storage TPV system. Furthermore, separating the cell from the radiator by transparent insulator layer or undoped semiconductor layer will help to protect the TPV cell from the hot radiator. It would also reduce the cell temperature by means of lower conduction and convection heat transfer to the cell.

### 8.3. Cost-Effectiveness of TPV Cell Commercialization

One of the major challenges in TPV application is to develop lower-cost of commercialized TPV cells. Some of the TPV structures are fabricated using expensive growth technology such as MBE. Additionally, most TPV cells require front and back surface field layers to optimize cell performance. These layers enhance the collection of long and short photogenerated carriers [242]. Hence, expensive epitaxial growth is employed in the deposition process of these layers.

Lattice-matched TPV substrate is another issue that makes the TPV production cost-ineffective. This is because the production of thick TPV substrate is expensive, which makes the fabrication of thin substrate in sub-micron scale a challenging issue. Therefore, the incorporation of commercially available and cost-effective substrates to grow TPV cells is a significant solution to address the high fabrication cost of TPV cells. For instance, growing a GaSb layer on GaAs substrate is a lower cost option as compared to GaSb substrate. Currently, ELOG method is used to grow GaSb junction on GaAs substrate with low mismatch defect [77]. Therefore, research on integrating ELOG method in different TPV materials will be an interesting research topic. Furthermore, the combination of Zn diffusion and epitaxial growth method was reported for InAsSbP/InAs p/n junction on InAs substrate by Krier et al. [65]. The combination allows higher cell performance due to low surface recombination. Further investigation for other types of TPV materials that could potentially reduce the economical barrier for mass scale TPV production is needed.

On top of that, Ge with a bandgap of 0.66 eV is a cost-effective candidate for the fabrication of TPV devices. Ge structure can be easily deposited on either Ge or GaAs substrates. However, the cell efficiency of Ge is lower than those of GaSb and InGaAs TPV cells. This is attributed to its higher density of states in the conduction band caused by the high electron mass as a consequence of high dark current, low $V_{oc}$ and high-temperature coefficient [202]. In addition, the quality of the Ge crystal with indirect bandgap tends to induce the recombination rate in the TPV cell, which decreases the overall efficiency. A number of studies have been conducted to improve the performance and to reduce the fabrication cost of Ge cell. For example, amorphous silicon (a-Si:H) was demonstrated to create a surface passivating material for Ge [243]. This structure generates cell efficiency, FF, $V_{oc}$ and *I_sc_* of 5.34%, 61.8%, 205.5 mV, and 42.1 mA/cm^2^, respectively. The investigation of P-i-N Ge cell and surface treatment on c-Ge using PH_3_ exposure shows significant improvement of $V_{oc}$ with better temperature coefficient [244,245]. Further studies are needed to improve the light absorption of Ge cell at thin absorber layer integrated with light-trapping techniques. It is worth noting that the high output power per unit area of TPV cells may offset the drawbacks related to their high cost, especially if the radiation temperature is high. For example, GaSb TPV under 1473 K radiation temperature reported an output power density of 0.82 W/cm^2^, which was almost 30 times higher than the output generated from the best GaAs solar cell [181].

### 8.4. Low TPV Cell Conversion Efficiency

**Figure 6:**
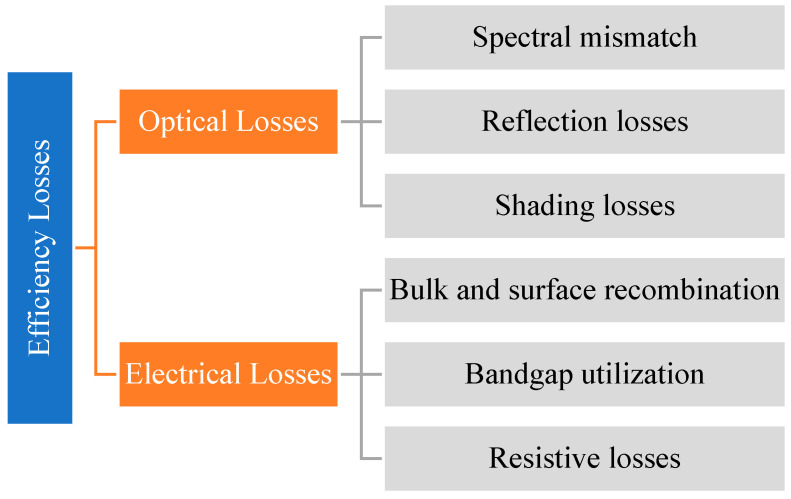
The TPV cells conversion efficiency losses.

The low conversion efficiency of TPV cells remains the key challenge in realizing viable TPV system. The TPV cells efficiency losses can be categorized into two main factors, that are optical and electrical losses, as depicted in Figure 6. In particular, the losses associated with spectral mismatch and bandgap utilization were emphasized in Section 8.1.

The prediction of optical-to-electrical conversion efficiencies via fundamental calculation in TPV is comparable to solar PV technology. However, lower optical-to-electrical conversion efficiency of the overall TPV system is observed in the real condition [246]. This is mainly due to the poor spectral control from the generator up to the TPV cell, resulting in serious optical losses which are associated with the reflection and shading losses. The reflection problem can be mitigated by implementing an ARC as well as back surface reflector, while the shading loss can be reduced by optimizing the metal coverage area on the cell active surface.

Other than that, electrical losses such as bulk and surface recombination are the influencing factors of the cell conversion efficiency. For instance, majority of the reported works on InGaAs cell optimization focused on optimizing the electrical issue of the cell and improving the *V_oc_*. This was achieved by reducing the thickness of the absorber layer. However, the cell needs to be optically thick to absorb most of the incident illumination. Generally, TPV illumination flux is usually shifted to infrared wavelengths, and thicker absorber is needed to improve the absorption of infrared radiation and significantly increase *J_sc_*. The use of intrinsic absorber layer in P-i-N or N-i-P TPV cells can increase *J_sc_*, and increase the ratio of generation to recombination as the generated carriers in i-layer has high mobility and lifetime. Therefore, to maximize the cell efficiency, both optical and electrical losses must be considered to obtain an optimized designed TPV cell.

On the other hand, resistive losses are related to the effectiveness of the metal contact, which allows smooth transportation of the photogenerated carriers to the external circuit. Although the importance of metal contact is highly recognized, the study of ohmic contact to semiconductor materials received less attention. For example, the process technology for the fabrication and metallization techniques needs to be further developed to facilitate a good metal contact formation onto the GaSb semiconductor material. Other than that, the optimization of TPV cell ohmic superconductive metal contact would ease the flow of high current density. This can be achieved by designing transparent metamaterial metal with ohmic work function.

### 8.5. Near-Field TPV System

Near-field TPV system is designed by separating the radiator from the TPV cell by only a few nanometres to centimetres gap distance [11]. The amount of power transferred significantly decreases with longer travelling distance, that can be presented by the inverse square law [164]. In nanometer gap distance, there will be near-field coupling, resulting in Super-Planckian characteristic. In recent years, rapid developments in the theoretical, computational, and experimental investigation of near-field TPV have taken place due to its ability to increase the amount of energy transfer into TPV converter. The near-field TPV is still in the early stages of modeling and simulation, where different materials and techniques are being investigated. For example, the characterization of multi-layer graphene on the top of InSb TPV cell [247] and the optimization for near-field TPV using a genetic algorithm method [248]. Near-field TPV system performs much better than far-field system for gap distance between 12 µm to 60 nm under low-radiation temperature (<655 K) [249,250]. Near-field was also implemented in hybrid thermophotonic-PV and thermionic-PV converters. While the former uses light-emitting diode at the hot side to produce an output power of 9.6 W/cm^2^ at 600 K radiator and 10 nm gap distance, the latter depends on both electrons and photons emission at the radiator [249,251]. Optimum near-field thermionic-PV converter has the potential to generate an output power density > 100 W/cm^2^ at 2000 K radiation temperature [252].

## 9. Conclusions

Thermophotovoltaic system shows great benefits as an energy source with a full-time operating regime of blackbody temperature 500–2000 K as compared to solar photovoltaic system. The two dominant TPV technologies, which are the gallium antimonide and indium gallium arsenide, were extensively reviewed due to the vast integration in both industries and research areas. The fabrication of both cells is conducted based on non-epitaxial growth such as zinc diffusion and ion implantation methods, as well as epitaxial growth through liquid phase epitaxy, metal-organic vapor phase epitaxy and molecular beam epitaxy techniques. Non-epitaxial method is the preferable option as the manufacture can be done with low cost and less tedious procedures. On the other hand, an epitaxial of ternary of quaternary layer is essential to reduce the bandgap of the cell for a thermal photovoltaic system with heat source temperature of less than 1000 K. The performances for both cells were summarized in terms of several parameters such as open-circuit voltage, short-circuit current, current density and the cell efficiencies. In addition, the improvement for the cell performances was recapped with structural and functional optimization such as metal contact, doping concentration, thickness, window, cap, buffer, surface field layers, graphene, metamaterial selective radiator and monolithic interconnected modules.

The practical applications for a thermal photovoltaic system include nuclear generator for space applications, hybrid electric vehicles, industrial and residential power supplies, waste heat recovery, solar thermophotovoltaic systems and portable electric generators. However, challenges in terms of spectral mismatching, internal cell temperature, and fabrication cost should be overcome to achieve a complete and more efficient thermophotovoltaic system. Hence, the key contribution of this study is the comprehensive analysis of gallium antimonide and indium gallium arsenide TPV cells to provide a tremendous outline on the improvement to the current achievement and their future deployment in the technology of energy and power system. The review has articulated some important and selective recommendations for the development of the TPV cells, and a few main research areas are listed below:

- One of the main challenges that degrade the performance of TPV system is caused by the mismatch between the TPV spectral and the cell bandgap. Since optimum cell performance is achieved when the majority of the blackbody spectrum is absorbed in the TPV cell, it is crucial to select the suitable narrow bandgap material based on the heat source temperature. Furthermore, the efficient reflection and recycling of sub-bandgap photons in the radiator will significantly enhance the conversion efficiency of the TPV system.
- The cooling system in the TPV structure is significant since the cells are located near the heat radiator and exposed to the high radiant energy. Cell temperatures higher than 300 K lead to performance deterioration, and a cooling system should be considered to sustain the performance of the TPV system. The effective cooling system would consume the minimum energy and will play an important role to boost the future of TPV technology. The integration of PCM can help to cool the TPV system without any energy consumption. Furthermore, separating the cell from the radiator using transparent insulator or undoped semiconductor layer will help to reduce the cell temperature.
- The investigation to reduce the cost of TPV cells. The use of ELOG fabrication which sacrifice layer between the junction and the substrate which allows the integration of cheap substrates such as Si, GaAs and InP. Further development on the fabrication methods would significantly enhance the TPV cells performance with respect to their cost. It is worth noting that Ge semiconductor material has lower cost as compared to other narrow bandgap materials. However, the Ge TPV cell is required to be optically thick, and further studies on light trapping techniques are required to improve the light absorption of Ge cell at a thin absorber layer.
- The investigation of TPV cell conversion efficiency. TPV cells operate at high radiation energy, and the development of cell with minimum electrical and optical losses would significantly improve the output performance of TPV cells. Research on structures optimization for near-field TPV spectrums would help to reduce the optical and electrical losses in the semiconductor layers. Furthermore, the development of effective metal contact will significantly ease the flow of high current density. This can be achieved by engineering the ohmic superconductive/transparent metamaterial metal with ohmic work function. The transparent metal will cover the entire surface of the structure, which reduces the crowding effect with no impact to the light absorption in the absorber layer.
- The development of the standard by which TPV cells are characterized, and the performances are determined. Previous reported works on TPV cells characterization vary in terms of the illumination intensity, the utilization of filter, and other measurement variations such as the variation of radiators, radiator temperature, cell temperatures, cavity geometry, and system scale, which makes the comparison of TPV cells a challenging task.

## Figures and Tables

**Table 1:** Characteristics of Different Growth Methods for TPV Cells.

Growth Method		Growth Rate	Heterojunction	Temperature	Vacuum (Y/N)	Safety	Cost	Absorb Thickness	Thickness Control (Y/N)	Absorb Doping	Doping Control (Y/N)
Epitaxial	MBE	0.082–0.278 nm/s [81,82,83]	Produces super lattice heterostructure structure [84,85]	723–808 K [80,86,87]	An ultra-high vacuum pressure lower than 5 × 10^−11^ Torr [84,88]	Toxic and required safety system for hydride gases [88]	Very expensive and complex [89]	2–10 µm [80,90,91,92]	Precise control [60,93]	8x10^16^–2x10^17^ [62,80,91,92,94]	Precise control over doping level and composition [60,93]
	MOVPE	0.0006–0.63 µm/s [93,95,96,97,98,99]	Suitable for heretojunction structure [93,96]	773–903 K [73,93,95,96,97,98,99,100]	Pressure ranging between 40–600 Torr [97,98,99,101]	Highly toxic	Expensive equipment and complex than LPE [89]	1–6 µm [96,97,102,103,104,105]	Precise control [93,100]	2.2 × 10^16^–5 × 10^17^ [96,97,102,103,104,105]	Doping and composition level are highly precise [93,100]
	LPE	2–15 µm/s 10 to 100 times faster than MOVPE or MBE [95,106,107]	Not very suitable	623–883 K [65,67,69,106,108,109]	Slightly above atmosphere pressure [65,69]	Produce non-toxic or less dangerous substances [108,109]	Simple and inexpensive method [108]	2–200 µm [69,107,109]	Less precise [84,95] but it can be improve with lower growth rate [110]	1 × 10^17^–5 × 10^18^ [70,109,111]	n/a
Non-Epitaxial	Diffusion	2–5 h to complete the diffusion [49,112]	Not suitable	693–753 K [62,113,114]	Diffusion closed box at vacuum level [49]	n/a	Simple and inexpensive	100–400 µm	n/a	3 × 10^17^–2.3 × 10^20^ [52,62,111]	Less precise [113]
	Ion implantation	n/a	Not suitable	80–373 K [115]	300–1000 Torr [116]	Ionized radiation	Less expensive [60]	100–400 µm	n/a	n/a	Precise control

**Table 2:** The performance comparison of Zn-diffused GaSb TPV cell.

Ref	Highlighted Condition	*T_rad_* (K)	*T_cell_* (K)	*V_oc_* (V)	*I_sc_* (A)	*J_sc_* (mA/cm^2^)	*FF* (%)	*P_max_* (W/cm^2^)	$\eta_{cell}$ (%)	Exp./ Pred.
[131]	Consists of shingle mounted 72 GaSb cells	1523	323	11.94	18.76	n/a	68.0	1.0	n/a	Exp.
	Performance assumed at cell temperature at 323 K	1533	323	n/a	n/a	n/a	n/a	n/a	15.4	Pred.
[133]	Consists of GaSb cells array connected in series in a conical TPV system	1673	n/a	1.77	1.05	n/a	66.4	n/a	n/a	Exp.
	Consists of GaSb cells array connected in parallel in a cylindrical TPV system	1673	n/a	0.41	3.5	n/a	62.4	n/a	n/a	
	Zn diffusion source using a polymer film layer	AM 1.5	n/a	0.5	n/a	38.56	66	n/a	n/a	
[134]	Consists of GaSb arrays of 4 × 3 cells	AM 1.5	n/a	1.69	3.55	n/a	64.1	3.8	4.5	Exp.
	Operated under W radiator	1900	n/a	~0.48	n/a	n/a	~70	n/a	19	
	Operated under SiC radiator		n/a	0.48	n/a	4.5	65	n/a	n/a	
[135]	SiC radiator with 1.1 cm radiator-cell distance	1223	325.2	0.34	2.29	n/a	n/a	0.26	n/a	Exp.
[49]	A silver metal was used to reduce fabrication cost	AM 1.5	n/a	0.281	n/a	29.0	48.5	n/a	3.9	Exp.
	Characterization on the JXC’s commercialised cell	AM 1.5	n/a	0.326	n/a	32.3	52.7	n/a	5.5	
[3]	Consists of 10 × 14 GaSb TPV cells circuit	1773	n/a	n/a	n/a	n/a	n/a	1.8	20	Pred.
		1573	n/a	n/a	n/a	n/a	n/a	1.1	18	Pred.
	Single cell measurement	1548	n/a	n/a	n/a	n/a	n/a	1.5	29	Exp.

Note: n/a means no data available in the literature. *T_rad_* is the radiation temperature under measure. Exp. stands for experimental data and Pred. means simulation or mathematical model. Collaboration work of JX Crystal Inc. and a research group in Hohai University, Nanjing, China published several papers on the enhancement of GaSb TPV cells [50,51,136]. In 2015, they reported that the performance of GaSb TPV cell can be improved using n-type emitters at the front-side of the TPV cell active area with PC1D software simulation [51]. In addition, a recent optimized design of a heterojunction on p-doped GaSb with hydrogenated amorphous Si interface passivation has been presented with the *IQE* surpasses 90% and power output density of 2 W/cm^2^ in the short-wave range under a blackbody radiation of 1500 K [136]. In principle, TPV cells operate at their optimum performance when GaSb energy bandgap is spectrally matched to the blackbody spectrum [4]. Therefore, the choice of utilizing the GaSb TPV cells is highly dependent on the source of radiation temperature or the types of radiators used in the system.

**Table 3:** The reported metal contact optimization work.

Ref	GaSb Layer Type	Doping Concentration	Metallization	Specific Contact Resistance	Metal Forming Process
[145]	p-type	8 × 10^17^ cm^−3^	Au	1 × 10^−4^ Ω cm^2^	Annealing process with T = 523–623 K
			AuZn	1 × 10^−5^ Ω cm^2^	
	n-type	n/a	Au, AuTe, AuSn	0.5–1 × 10^−4^ Ω cm^2^	Annealing process with T = 523 K
[146]	p-type	8 × 10^16^ cm^−3^–1 × 10^19^ cm^−3^	Au, Au(Zn), Au(In, Zn), Au(Ge), Ag(Sn), Ag(In), In, In(Zn), In(Ge) and Al	~5 × 10^−5^ Ω cm^2^ for doping 10^18^ cm^−3^ ~5 × 10^−6^ Ω cm^2^ for doping 1 × 10^19^ cm^−3^	Annealing process with T = 523–623 K for 10–30 min
[147]	n-type	1.2 × 10^18^ cm^−3^	Pd/Ge/Au/Pt/Au	1 × 10^−5^ Ω cm^2^	RTA at T = 543 K for 60 s
[141]	n-type	5 × 10^18^ cm^−3^–1 × 10^19^ cm^−3^	(Pd+Mo)/Ge/Au/Pt/Au	3 × 10^−6^ Ω cm^2^	RTA at T = 573 K for 45 s
			Ge/Au/(Pd+Mo)/Pt/Au	2 × 10^−4^ Ω cm^2^	RTA at T = 553 K for 45 s
[138]	n-type	5 × 10^17^ cm^−3^–1 × 10^19^ cm^−3^	Ni/Ge/Au/Pt/Au, Pd/Ge/Au/Pt/Au, Ge/Au/Ni/Pt/Au, Ge/Au/Pd/Pt/Au	1 × 10^−2^ Ω cm^2^–1 × 10^−6^ Ω cm^2^	RTA at T = 533–593 K for 45 s

**Table 4:** Performance comparison of In_x_Ga_1-x_As-based TPV Cells.

Structure	Growth Method	*T_rad_*	Suns	*T_cell_* (K)	*λ_c_* (µm)	Area (cm^2^)	*V_oc_* (V)	*J_sc_* (mA/cm^2^)	*FF* (%)	*J_0_* (A/cm^2^)	*P_max_* (mW/cm^2^)	*η* (%)	*EQE_peak_* (%)@ *λ*(µm)	*IQE_peak_* (%)@ *λ*(µm)	Ref
In_0.53_Ga_0.47_As	MOVPE	AM 1.5	1	n/a	1.7	n/a	0.35	57.7	71.2	n/a	n/a	14.37	~1	n/a	[103]
In_0.53_Ga_0.47_As	MOVPE	AM 1.5	1	n/a	1.7	0.032	0.39	35.2	71	n/a	n/a	9.72	~1	n/a	[102]
In_0.58_Ga_0.42_As	MOVPE	Tungsten–halogen (3250 K)	_	303	1.7	n/a	0.405	28.2	69	n/a	n/a	12.4	~90 (1.3)	~99 (0.9)	[164]
In_0.62_Ga_0.48_As	MOVPE		_	303	1.7	n/a	0.419	28.8	65	n/a	n/a	12.1	~90 (1.3)	~99 (0.9)	[164]
In_0.53_Ga_0.47_As	MOVPE	AM 1.5	1	300	1.7	n/a	0.360	42	n/a	6.45 µ	n/a	12.33	n/a	n/a	[159]
In_0.53_Ga_0.47_As	MOVPE	1323 K	_	300	1.7	n/a	0.22	0.6	57.5	0.067µ	0.06	16.4	n/a	n/a	[159]
In_0.53_Ga_0.47_As	LPE and diffusion	Tungsten 1800 K	_	n/a	1.7	1	0.465	1000	64	n/a	n/a	~15	90 (0.8)	n/a	[52]
In_0.53_Ga_0.47_As	MOVPE	AM 0	1	298	1.65	0.09	0.333	39.73	71.6	n/a	n/a	13.3	60 (1.4)	n/a	[98]
In_0.53_Ga_0.47_As	–	4000 K	_	300	1.7	0.1	0.38–0.44	50–600	69–72.5	n/a	n/a	15–18	n/a	n/a	[170]
In_0.53_Ga_0.47_As	MOVPE	3300 K	_	300	1.7	0.01	0.291–0.315	181–390	39–40	n/a	20.7– 49.4	4.14 –9.88	n/a	n/a	[171]
In_0.68_Ga_0.32_As	MOVPE	AM 1.5	1	300	~2	n/a	0.23	56	n/a	1362 µ	n/a	8.06	n/a	n/a	[159]
In_0.68_Ga_0.32_As	MOVPE	1323 K	_	300	~2	n/a	0.175	10	55	11.6µ	n/a	19.1	n/a	n/a	[159]
In_0.69_Ga_0.31_As	MOVPE	AM 1.5	1	n/a	2.10	n/a	0.215	47.6	57	n/a		6.9	~1 (80)	n/a	[156]
In_0.69_Ga_0.31_As	MBE	2050 K	_	298	~2	2.7 × 10^−2^	0.357	1180	68.1	n/a	0.2095	n/a	~67 (1.4)	n/a	[91]
In_0.68_Ga_0.32_As	MOVPE	AM 1.5	1	300	~2	0.0625	0.19–0.23	43–56	n/a	n/a	n/a	5.31 –8.06	n/a	n/a	[155]
In_0.69_Ga_0.31_As	N/A	AM 0	5.7–27.2	298	~2	0.13	0.27–0.38	45	~66.3 −71.3	443 n	(0.13–3.42) × 10^2^	5.4–7.8	75 (1.5)	n/a	[166]
In_0.69_Ga_0.31_As	MBE	1920 K–2050 K	_	298	~2.1	2.82 × 10^−2^	0.334–0.355	(1.11–2.26) × 10^3^	65.6–66.5	7.2 ×10^−4^–3.3 x10^−5^	0.195–0.532	n/a	100 (1.2)	n/a	[85]
In_0.68_Ga_0.32_As	MOVPE	infrared lamp illumination	0 _ 72 V	293	~2.05	n/a	0.38	1.7 × 10^3^	70	n/a	0.25 × 10^3^	n/a	82 (1.2)	n/a	[73]
In_0.72_Ga_0.28_As	MOVPE	tungstenhalogen light	_	n/a	~2.2	1	0.291–0.301	(3–3.7) × 10^3^	n/a	0.07–1	n/a	n/a	56.7 (2)	n/a	[172]
In_0.72_Ga_0.28_As	n/a	1273–1773 K	15 Wcm^−2^	300	~2.2	n/a	0.317–0.332	(3.87–7.08) × 10^3^	48.7–57.7	n/a	0.71–1.15	4.7–7.6	n/a (1.9)	n/a	[163]
In_0.72_Ga_0.28_As	n/a	n/a	n/a n/a	300	~2.2	1	0.28–0.32	(1–4) × 10^3^	54–65	2 × 10^−5^	0.18–0.58	n/a	88 (1.6)	n/a	[173]
In_0.72_Ga_0.28_As	MOVPE	n/a	n/a	n/a	~2.2	1	0.298 –0.31	(1.1–1.2) × 10^3^	62–65	n/a	n/a	n/a	~70 (1.5)	n/a	[174]
In_0.78_Ga_0.22_As	PECVD	1273 K	n/a	298	2.4	n/a	0.307	3.65 × 10^3^	59.8–63.2	56–1330 µ	n/a	n/a	n/a	~2.2 (99)	[175]
In_0.79_Ga_0.21_As	MOCVD	1273 K	n/a	298	~2.5	1	38	26.3	n/a	n/a	0.2	n/a	n/a	n/a	[176]
In_0.83_Ga_0.17_As	MBE	n/a	n/a	300	~2.65	n/a	n/a	3.7 × 10^−3^	n/a	n/a	n/a	n/a	n/a	n/a	[177]

## Data Availability

The data presented in this study are available on request from the corresponding author.

## Appendix A

**Short Name**	**Full Name**	**Symbol**	**Unit**	**Meaning**
TPV	Thermophotovoltaic	*P_heat source_*	W/cm^2^	Power of heating source
GaSb	Gallium antimonide	*P_source loss_*	W/cm^2^	Source heat loss
InGaAs	Indium gallium arsenide	*P_radiant_*	W/cm^2^	The radiant power
Ge	Germanium	*P_gap_*	W/cm^2^	The bandgap power
InAs	Indium arsenide	*P_gap,loss_*	W/cm^2^	The bandgap power loss
InGaAsSb	Indium gallium arsenide antimonide	*P_recuperate_*	W/cm^2^	The recuperated power
InAsSbP	Indium arsenide antimonide phosphide	*P_out_*	W/cm^2^	Output power
InGaAsSbP	Indium gallium arsenide antimonide phosphide	*η*	%	Cell efficiency
TE	Thermoelectric	*P_in_*	W/cm^2^	Input optical power
SiC	Silicon carbide	*J_sc_*	A/cm^2^	Short-circuit current density
W	Tungsten	*V_oc_*	V	Open-circuit voltage
PhC	Photonics crystal	*I_sc_*	A	Short-circuit current
YZA	Yttria-stabilised zirconia/alumina composite	*FF*	%	Fill factor
HEV	Hybrid electric vehicle	*T_cell_*	K	Cell temperature
PCB	Pseudo-closed box	*J_o_*	A/cm^2^	Dark saturation current density
RTA	Rapid thermal annealing	*n*		Ideality factor
LPE	Liquid phase epitaxy	*J_ph_*	A/cm^2^	Photocurrent density
ELOG	Epitaxial lateral overgrowth	*J*	A/cm^2^	Current density at the load
VPE	Vapour phase epitaxy	*IV*	AV	Current-voltage
MBE	Molecular beam epitaxy	*e_o_*	coulomb	Charge
MOVPE	Metal-organic vapour phase epitaxy	*J_m_*	A/cm^2^	Maximum current density
PECVD	Plasma-enhanced chemical vapour deposition	*V_m_*	V	Maximum voltage
Ni	Nickel	*P_m_*	W/cm^2^	Maximum power
Pd	Palladium	*R_L_*	Ω	External load
Au	Gold	*EQE*	%	External quantum efficiency
Pt	Platinum	*λ*	m	Wavelength
Mo	Molybdenum	*Φ(λ)*	m^−2^s^−1^	Incident photon flux
TCO	Transparent conductive oxide	*d(λ)*	m	Penetration distance
Si:H	Hydrogenated amorphous silicon interface	*R*(*λ*)	%	Reflection as a function of wavelength
MPhCs	Metallic photonic crystal	*P_(x)_*	A/cm^2^	Power received by the TPV cell
GaAs	Gallium arsenide	*IQE*	%	Internal quantum efficiency
InP	Indium phosphide	*η_TPV_*	%	Efficiency of TPV system
NREL	National Renewable Energy Laboratory	*η_sp_*	%	Spectral efficiency
SRH	Shockley-Read-Hall	*E*(*λ*)	W/cm^2^. µm	Blackbody spectrum
MIMs	Monolithic Interconnected Modules	*ε*(*λ*)	n/a	Spectral emissivity
InGaSb	Indium gallium antimonide	*E_g_*	eV	Bandgap energy
InSb	Indium antimonide	*λ_c_*	m	Cut-off wavelength
InAsSb	Indium arsenide antimonide	*λ_p_*	m	Peak emissivity
STPV	Solar TPV systems	*n_i_*	cm^−3^	Intrinsic concentration
CHP	Combined Heat and Power	*QE*	%	Quantum Efficiency
WHR	Waste Heat Recovery			
PCM	Phase change materials			
a-Si:H	Amorphous silicon			
